# Clonally selected primitive endothelial cells promote occlusive pulmonary arteriopathy and severe pulmonary hypertension in rats exposed to chronic hypoxia

**DOI:** 10.1038/s41598-020-58083-7

**Published:** 2020-01-24

**Authors:** Aneel R. Bhagwani, Daniela Farkas, Brennan Harmon, Kayla J. Authelet, Carlyne D. Cool, Martin Kolb, Elena Goncharova, Mervin C. Yoder, Matthias Clauss, Robert Freishtat, Laszlo Farkas

**Affiliations:** 1 Department of Internal Medicine, School of Medicine, Virginia Commonwealth University, Division of Pulmonary Disease and Critical Care Medicine, Richmond, VA USA; 2 Department of Physiology and Biophysics, School of Medicine, Virginia Commonwealth University, Richmond, VA USA; 3 Department of Internal Medicine, Division of Pulmonary, Critical Care & Sleep Medicine, College of Medicine, the Ohio State University, Columbus, OH USA; 4 Department of Pediatrics, Division of Emergency Medicine, Children’s National Health System, Washington, DC USA; 5 Department of Pathology, University of Colorado Denver, Denver, CO USA; 6 Departments of Medicine, Pathology and Molecular Medicine, McMaster University, Hamilton, ON Canada; 7 Department of Medicine, Division of Pulmonary, Allergy and Critical Care, Vascular Medicine Institute, and Department of Bioengineering, University of Pittsburgh, Pittsburgh, PA USA; 8 Center for Regenerative Medicine and Engineering, Indiana University, Indianapolis, IN USA; 9 Pulmonary, Critical Care, Sleep & Occupational Medicine, Indiana University, Indianapolis, IN USA

**Keywords:** Experimental models of disease, Preclinical research, Translational research, Molecular medicine

## Abstract

One current concept suggests that unchecked proliferation of clonally selected precursors of endothelial cells (ECs) contribute to severe pulmonary arterial hypertension (PAH). We hypothesized that clonally selected ECs expressing the progenitor marker CD117 promote severe occlusive pulmonary hypertension (PH). The remodelled pulmonary arteries of PAH patients harboured CD117^+^ ECs. Rat lung CD117^+^ ECs underwent four generations of clonal expansion to enrich hyperproliferative ECs. The resulting clonally enriched ECs behaved like ECs, as measured by *in vitro* and *in vivo* angiogenesis assays. The same primitive ECs showed a limited ability for mesenchymal lineage differentiation. Endothelial differentiation and function were enhanced by blocking TGF-β signalling, promoting bone morphogenic protein (BMP) signalling. The transplantation of the EC clones caused arterio-occlusive PH in rats exposed to chronic hypoxia. These EC clones engrafted in the pulmonary arteries. Yet cessation of chronic hypoxia promoted lung cell apoptosis and resolution of vascular lesions. In conclusion, this is to the best of our knowledge, the first report that clonally enriched primitive ECs promote occlusive pulmonary arteriopathy and severe PH. These primitive EC clones further give rise to cells of endothelial and mesenchymal lineage as directed by BMP and TGF-β signaling.

## Introduction

Pulmonary arterial hypertension (PAH) remains a devastating disease with dismal prognosis. The pulmonary arteries of PAH patients have lesions of varying severity including formation of neointima, which obstructs the normal blood flow, and complex multicellular plexiform lesions. A current concept suggests that dysregulated programmed cell death, or apoptosis of ECs causes unchecked proliferation of a subset of apoptosis-resistant ECs^1,2^. The ECs retain a hyperproliferative and apoptosis-resistant phenotype even in culture^3^. These abnormal ECs in the plexiform lesions of PAH patients may originate from primitive endothelial-like stem cells by clonal selection^4^. This concept of primitive cells as source of aberrant ECs in PAH is supported by the findings of markers of primitive stem-like cells in the lung vascular lesions^5–7^. Likewise, hyperproliferative pulmonary artery smooth muscle cells may also be derived from progenitor cells in PAH^8,9^. During vascular development, primitive ECs give rise to a bipotent mesodermal source of definitive ECs and mesenchymal progenitor cells^10,11^. Generation of mesenchymal progenitor cells requires endothelial-to-mesenchymal transition (EnMT) and transforming growth factor-β (TGF-β) is an important driver of EnMT^12^. In addition, reduced expression of bone morphogenic protein receptor 2 (BMPR2) and reduced BMP signalling promote EnMT, suggesting that the balance between TGF-β and BMP signalling determines the phenotype of primitive ECs^13^. Primitive ECs are found in the vasculature of different adult organ systems, and these primitive ECs may be the source of highly proliferative endothelial progenitors and clonally expandable stem-like ECs, but also mesenchymal progenitor cells in the adult pulmonary vasculature^14–17^. Clonal expansion further contributes to neovascularization after ischemia in the systemic circulation^18^. The primitive, stem cell-like ECs also expressed CD117, a marker of primitive hematopoietic and endothelial cells and incidentally, CD117^+^ lesion cells occur in the occlusive pulmonary arteriopathy in human PAH and severe pulmonary hypertension (PH) induced by chronic hypoxia and SU5416 in rats^5,17,19,20^.

We hypothesized that clonal selection of CD117^+^ ECs yields primitive EC clones that exhibit extensive proliferation, angiogenic sprouting, and the ability to give rise to definitive endothelium and mesenchymal cells depending on the balance between TGF-β and BMP signaling. We further hypothesized that transplantation of these clonally selected ECs promotes occlusive pulmonary arteriopathy and PH.

Primitive CD117^+^ ECs were isolated from the lung periphery of rats with ubiquitous expression of enhanced green fluorescent protein (EGFP). Four generations of clonal expansion led to detection of primitive CD117^+^ ECs that showed extensive proliferation, endothelial function, but also differentiated into mesenchymal lineages when switching growth medium and stimulating with TGF-β1. Transplantation of these primitive CD117^+^ EC clones, which will be referred to as “EC clones” throughout the manuscript, caused occlusive pulmonary arteriopathy and exaggerated PH in rats exposed to chronic hypoxia. The occlusive arteriopathy resolved spontaneously after cessation of chronic hypoxia with evidence of increased apoptosis in the lung tissue. Hence, our results indicate that primitive, clonally selected lung CD117^+^ ECs promote occlusive pulmonary arteriopathy in rats under exposure to chronic hypoxia.

## Results

### CD117^+^ endothelium is present in human PAH lungs

**Figure 1:**
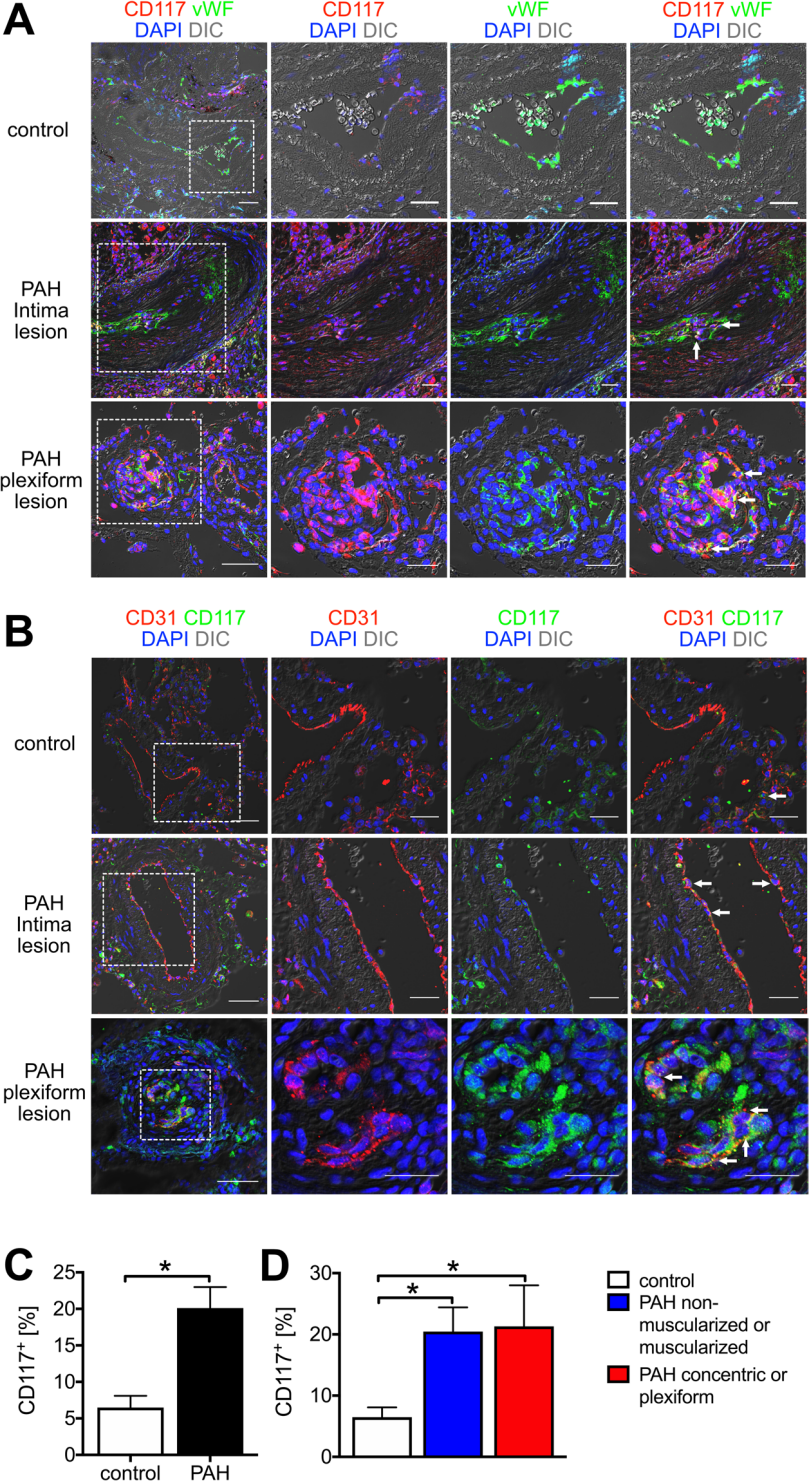
Localization of CD117^+^ ECs in PAH and control pulmonary arteries. (**A**,**B**) Representative pseudo coloured optical sections obtained by confocal microscopy and merged with differential interference contrast (DIC). (**A**) Occasional vWF^+^ cells (green pseudo colour) with less intense CD117 staining (red pseudo colour) were detected in the endothelial layer of pulmonary arteries from control subjects, whereas pulmonary arteries with intima lesions and particularly with plexiform lesions had substantial amounts of CD117^+^ vWF^+^ cells. (**B**) Similarly, CD117^+^ (green pseudo colour) CD31^+^ (red pseudo colour) cells were rare in the intima of pulmonary arteries of controls subjects, whereas multiple CD117^+^ CD31^+^ cells were found in pulmonary arteries from PAH patients (arrows). In control subjects, occasional CD117^+^ CD31^+^ cells were detected among the alveolar capillary cells (arrow). In (**A**,**B**), the image on the left shows an overview, and the images on the right demonstrate the area indicated with a dotted square in more detail. Scale bars: 50 μm (overview), 25 μm (detail). Counterstaining with DAPI (blue pseudo colour). (**C**,**D**) Quantification of CD117^+^ cells in pulmonary arteries of control subjects and PAH patients, in (**C**) summary of all pulmonary arteries is shown for each group, whereas (**D**) differentiates between non-muscularized/muscularized pulmonary arteries and pulmonary arteries with concentric or plexiform lesions in PAH patients. Note that total CD117^+^ cells and not CD117^+^ ECs were quantified. n = 3 (control), 6 (PAH). *P < 0.05.

These experiments were based on description of CD117^+^ cells in human PAH lesions^5^ and our previous observations of CD117^+^ vWF^+^ cells in the occlusive lung vascular lesions of rats treated with chronic hypoxia and SU5416^20^. We performed double IF staining to identify the *in situ* localization of CD117^+^ ECs in PAH lung vascular lesions. While pulmonary arteries from control subjects revealed rare CD117^+^ vWF (von Willebrand Factor)^+^, CD117^+^ CD31^+^ or CD117^+^ podocalyxin (PODXL)^+^ cells with faint CD117 staining, multiple CD117^+^ vWF^+^, CD117^+^ CD31^+^ or CD117^+^ PODXL^+^ cells were detected in the occlusive pulmonary arterial lesions of PAH patients, particularly in plexiform lesions (Fig. 1 and Supplemental Fig. S1). Overall, the fraction of CD117^+^ cells was increased in pulmonary arteries from patients with PAH and there was no difference between the fraction of CD117^+^ cells in PAH non-muscularized/muscularized pulmonary arteries and concentric/plexiform lesions (Fig. 1C,D). Hence, our findings confirm the augmented presence of CD117^+^ ECs in PAH pulmonary arteries with histological evidence of occlusive arteriopathy.

### Clonal expansion of rat lung CD117^+^ ECs

**Figure 2:**
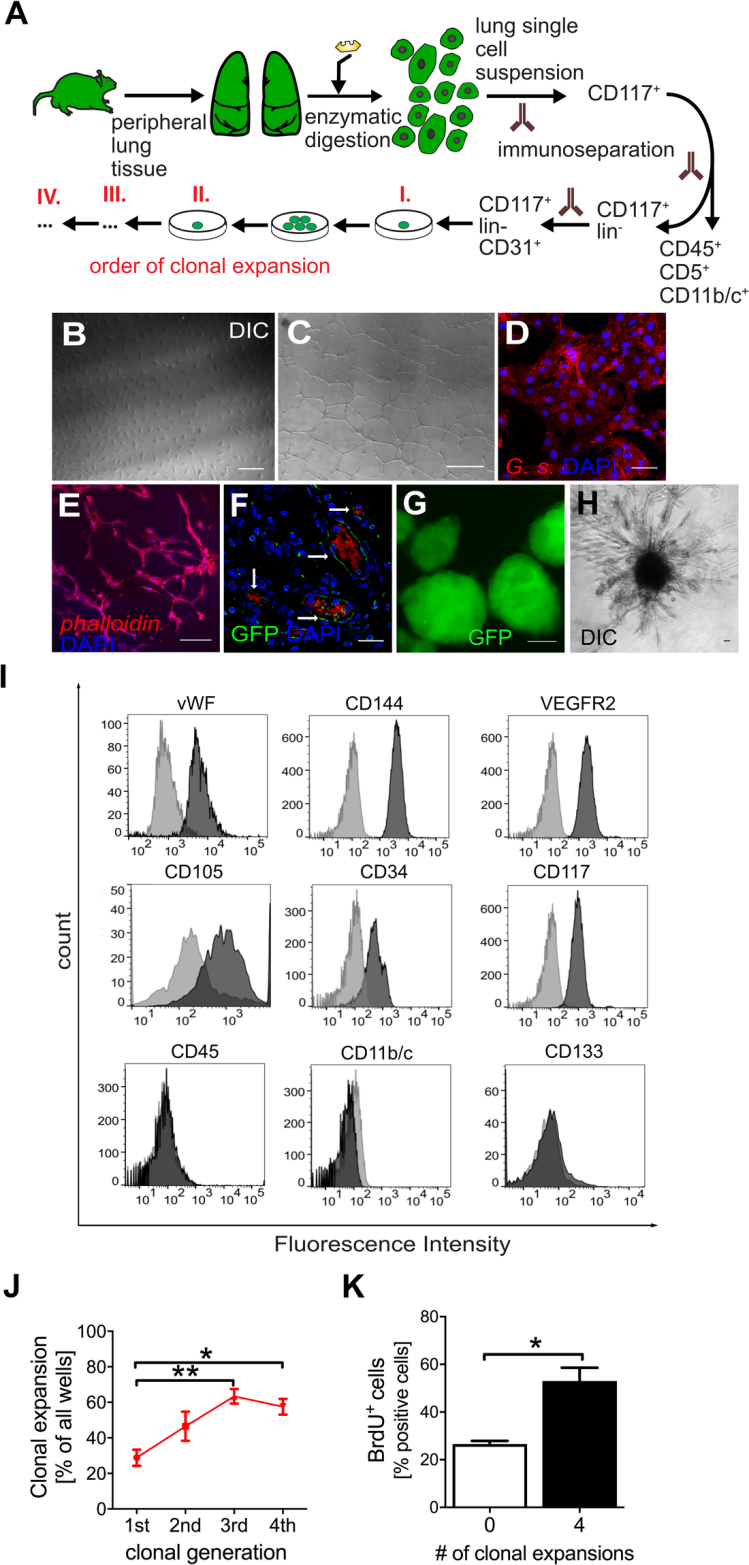
Isolation and characterization of EC clones from CD117^+^ ECs. (**A**) Isolation and clonal enrichment flow diagram for EC clones from the lungs of EGFP^+^ rats. (**B**) Representative differential interference contrast (DIC) image shows the typical endothelial cobblestone morphology of EC clones. (**C**) Representative DIC image of 2D matrigel assay. (**D**) Representative optical section (confocal microscopy) demonstrate binding of *Griffonia simplicifolia* lectin (red pseudo colour) in EC clones, indicating a microvascular phenotype (scale bar 25 μm). (**E**) 24 h 3D tube formation assay in fibrin shows formation of tube networks by EC clones (scale bar 200 μm). Cells were visualized by phalloidin staining of actin filaments (red pseudo colour). (**F**) confocal imaging of Matrigel plug at day 14 with EC clones stained for GFP (green pseudo colour) demonstrating GFP^+^ blood vessels. The red autofluorescence demonstrates blood and indicates active perfusion of the GFP^+^ vascular structures after 14 days. Scale bar: 25 μm. Counterstaining in (**D**–**F**) with DAPI. (**G**,**H**) Representative images demonstrate that EC clones form free-floating spheroids in low adhesion cell culture wells. When overlaid with a 50Vol%/50Vol% EGM2/Matrigel mixture, EC clone spheroids underwent angiogenic sprouting. The image (**G**) shows green fluorescence channel of EC clone spheroids (green pseudo colour), whereas image (**H**) shows DIC brightfield image of an EC clone spheroid after 7 days of sprouting. Scale bars: 50 μm. (**I**) Representative flow cytometry analysis of EC clones shows expression of endothelial markers (vWF, CD144, VEGFR2, CD105, CD34) and CD117, but not of typical hematopoietic and myeloid markers (CD45, CD11b/c and CD133). Note that vWF is an intracellular marker and required permeabilization of the cells during the staining process. (**J**) The fraction of clonally expandable wells increases from clonal generation 1 to 3. n = 3 (1st) and 6 (2nd, 3rd, 4th). (**K**) 4th generation EC clones proliferate faster than non-expanded CD117^+^ ECs (n = 3–4). *P < 0.05, **P < 0.01.

To expand on previous concepts of clonal expansion of ECs as a pathogenic process during PAH lesion development and our findings of CD117^+^ ECs in the lung vascular lesions, we tested whether clonally expanded CD117^+^ ECs contribute to development of occlusive lung vascular lesions. For this purpose, we employed globally EGFP expressing transgenic rats. After isolation of CD117^+^ lin^−^ CD31^+^ primitive ECs from the periphery of EGFP^+^ rat lungs (Fig. 2A), we performed four generations of clonal expansion *in vitro*, obtaining in each generation the largest colonies with endothelial appearance (Fig. 2A). The resulting CD117^+^ EC clones exhibited endothelial cobblestone morphology and generated networks of angiogenic tubes in 2D matrigel assays (Fig. 2B,C). CD117^+^ EC clones further bound *Griffonia simplicifolia* (*G*.*s*.) lectin, indicating a microvascular endothelial phenotype in the lung (Fig. 2D). EC clones were able to generate tubes in a 3D fibrin assay and formed GFP^+^ perfused blood vessels in *in vivo* matrigel plug assays (Fig. 2E,F). EC clones were further able to form 3D spheroids without attachment and these spheroids generated angiogenic sprouting when embedded into in a matrigel-media mixture (Fig. 2G,H). In addition, they expressed typical endothelial markers vWF, CD144, vascular endothelial growth factor receptor 2 (VEGFR2), CD105, CD34, but not common hematopoietic and myeloid surface markers CD45, CD11b/c and CD133 (Fig. 2I). As expected, clones derived from CD117^+^ lin^−^ CD31^+^ cells expressed CD117 (Fig. 2I). The fraction of expandable clones increased until the 3^rd^ clonal generation and remained high during the 4^th^ clonal generation (Fig. 2J). 4^th^ generation EC clones showed higher proliferation than not clonally expanded CD117^+^ ECs (Fig. 2K). ECs derived from the CD117^−^ cell pool showed typical functional endothelial characteristics, such as angiogenic network formation in 2D matrigel assays and 3D fibrin assays, binding of *G*.*s*. lectin (microvascular phenotype) and expression of endothelial markers (Supplemental Fig. S2). These cells were used as comparison for the *in vivo* experiments. To further identify the role of CD117 for the angiogenic properties of the CD117^+^ EC clones *in vitro*, EC clones were treated with a neutralizing antibody targeting the CD117 ligand stem cell factor (SCF). Blocking stem cell factor reduced cell viability/metabolism and angiogenic tube formation (Supplemental Fig. S3), supporting an important role for SCF/CD117 signalling.

### Mesenchymal lineage differentiation potential and the role of TGF-β

Because primitive ECs are a source of mesenchymal lineages during lung development and in PAH occlusive vascular lesions^11,21^, we tested whether the EC clones give rise to cells of mesenchymal lineage. When we switched EC clones from endothelial growth media (EGM-2MV) to minimal essential medium (MEM), we observed phenotypic changes towards an elongated, mesenchymal phenotype, which was associated with upregulation of smooth muscle cell markers *Acta2*, *Cnn1* and *Tagln* (Supplemental Fig. S4A,B). Interestingly, there was no significant loss of EC marker expression. To promote the differentiation towards a smooth muscle-like phenotype, the EC clones were treated with TGF-β1 and PDGF-B in MEM, and this treatment induced the expression of transcription factors *Snai1*, *Snai2* and *Twist1*, which drive endothelial-to-mesenchymal transition (EnMT)^21,22^. However, there was a downregulation of *Acta2* and *Tagln*, which is consistent with a proliferative switch in response to PDGF-B^23^. Stimulation with TGF-β alone in MEM medium lead to a trend towards elevated *Vcam1* expression, and a further increase in mesenchymal cell markers *Acta2*, *Cnn1*, *Tagln*, as well as mesenchymal transition transcription factor *Snai1* (Supplemental Fig. S4C,D).

We then tested whether adipogenic and osteogenic differentiation can be induced in the EC clones. In contrast to rat BM-MSCs, only few cells of EC clones survived adipogenic differentiation medium and lipid droplets were not observed (Supplemental Fig. S5A). However, we identified areas of Calcium deposition in EC clones after inducing osteogenic differentiation (Supplemental Fig. S5B). Hence, the CD117^+^ EC clones have the ability to differentiate into selected mesenchymal lineages, such as smooth muscle-like and osteoblast-like cells.

### ALK5 inhibition promotes angiogenesis and reduces mesenchymal transition in EC clones cultured in smooth muscle cell growth medium

**Figure 3:**
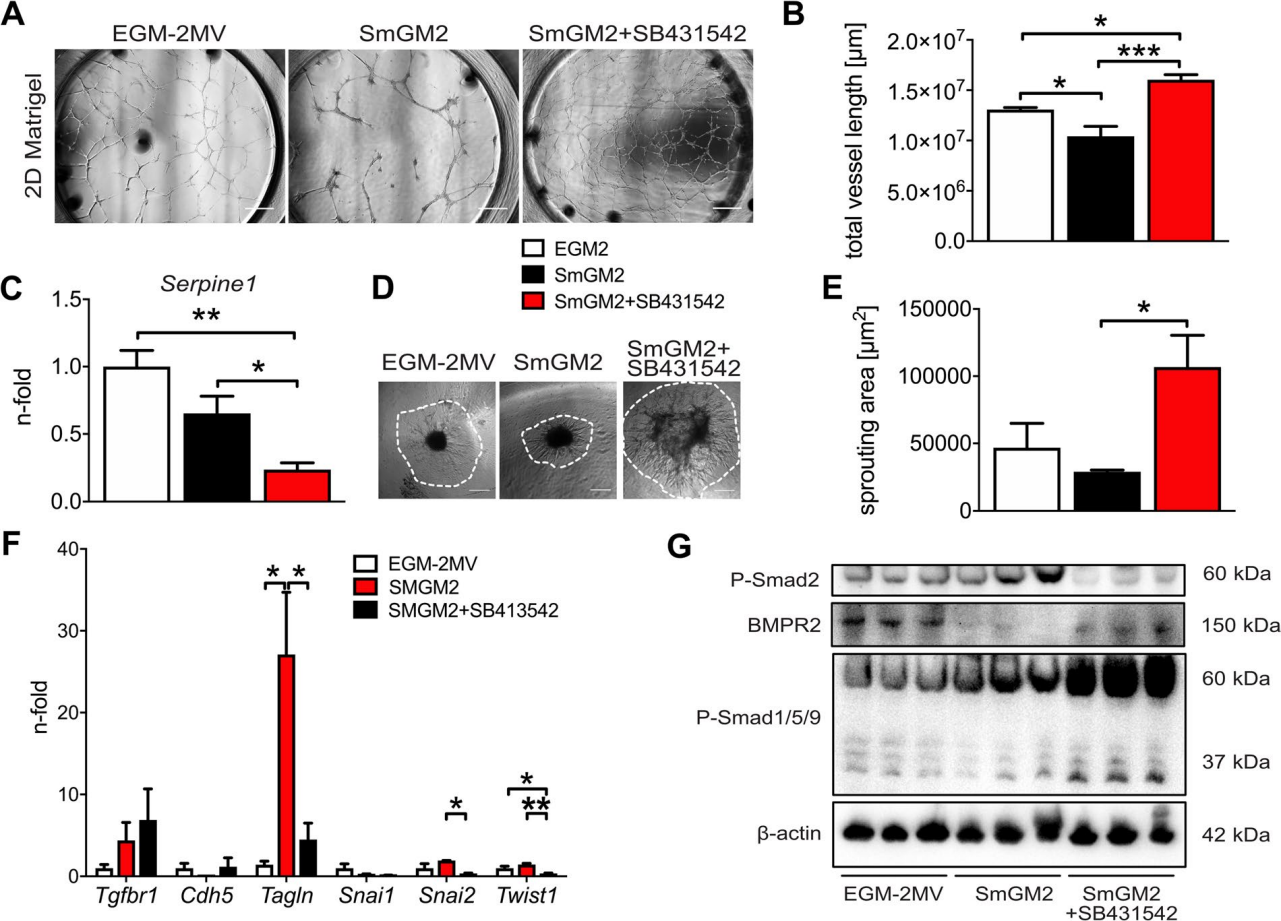
Transformation of EC clones to smooth muscle-like cells and angiogenic effect of ALK5 inhibition. (**A**) Representative software-stitched images of 24 h of 2D Matrigel assay (growth factor-reduced Matrigel) after 3 days of culture of EC clones in EGM-2MV, SmGM2 and SmGM2+SB431542 (10 μM). The ALK5 inhibitor SB431542 promoted tube formation. Scale bar: 500 μm. (**B**) Quantification of total tube length in EC clones grown in EGM-2MV, SmGM2 and SmGM2+SB431542. n = 5–6 per group (**C**) qRT-PCR for Serpine1 demonstrates inhibition of TGF-β pathway using SB431542. n = 7–8 per group. (**D**) Representative DIC images of spheroids sprouted for 72 h in a 50% Matrigel/50% growth media mixture for EGM-2MV, SmGM2 and SmGM2+SB431542 groups. The dotted lines demonstrate the boundaries of the sprouting area used for quantification. Scale bar: 200 μm. (**E**) Sprouting area for EGM-2MV, SmGM2 and SmGM2+SB431542 groups. N = 5–7 per group (**F**) Gene expression profile indicates increased expression *of* mesenchymal markers and mesenchymal transition transcription factors in SmGM2 medium, which is prevented by SB431542. n = 3 per group. *P < 0.05, **P < 0.01, ***P < 0.001. (**G**) Representative Western blot analysis of phospho(P)-Smad2, BMPR2 and P-Smad1/5/9 in EC clones cultured in EGM-2MV, SmGM2 and SmGM2+SB411542. β-actin was used as loading control.

To further identify whether blocking TGF-β signalling promotes endothelial function and differentiation in non-endothelial growth media, we tested whether inhibition of the TGF-β type I receptor ALK5 promotes endothelial function by using 2D Matrigel tube formation assays and 3D spheroid sprouting assays. We found that whereas culturing EC clones in smooth muscle growth medium 2 (SmGM2) decreased angiogenic tube formation and sprouting, ALK5 inhibition with SB431542 increased total tube length (2D Matrigel), reduced TGF-β downstream signalling (expression of the Smad2/3 transcriptional target *Serpine1*) and augmented sprouting area from 3D spheroids (Fig. 3A–E). In addition, the spheroids were irregular and larger under ALK5 inhibition as compared to the more regular spheroids when cultured in EGM2 or SmGM2 without SB431542. Whereas SmGM2 promoted a mesenchymal phenotype in the gene expression pattern, concomitant treatment with SB431542 prevented this effect (Fig. 3F). In addition, SmGM2 promoted Smad2 phosphorylation, but reduced BMPR2 expression and Smad1/5/9 phosphorylation. In contrast, SmGM2 + SB431542 treatment reduced Smad2 phosphorylation, but increased BMPR2 expression and Smad1/5/9 phosphorylation (Fig. 3G). In conclusion, ALK5 inhibition prevents mesenchymal transition and enhances angiogenesis in CD117^+^ EC clones by favouring BMPR2/Smad1 signalling.

### Gene expression profiling of EC clones *in vitro*

**Figure 4:**
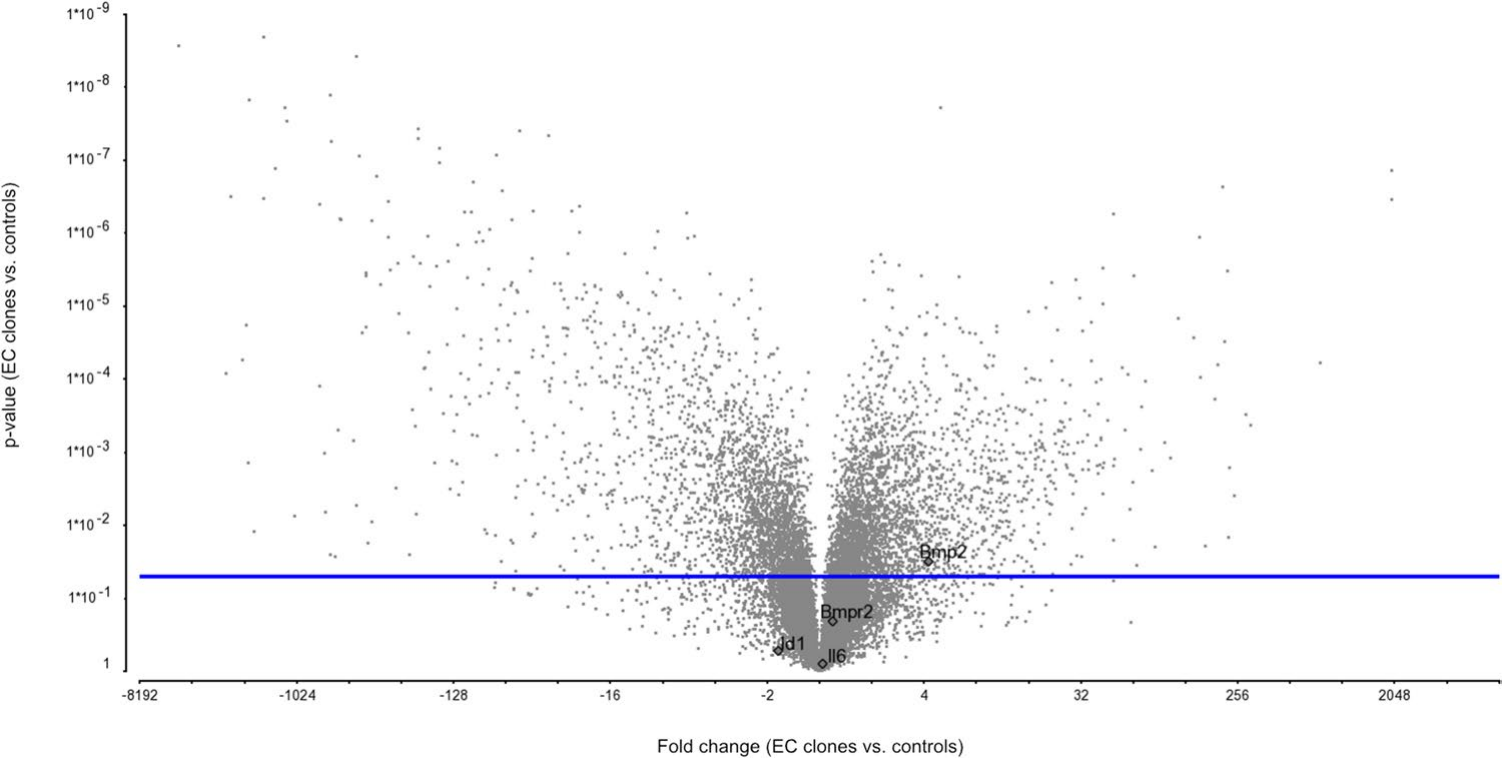
Transcriptome of EC clones vs. control ECs. Volcano plot of microarray data from 3 control ECs (ECs isolated from the CD117^−^ cell pool) and 4 EC clones show categorical differences of gene expression in 468 genes (*P* < 0.05 with FDR, fold change >10) between the two groups. This plot shows the correlation between fold change of expression of CD117^+^ EC clones vs. CD117^−^ ECs (x-axis) and P-value (y-axis), summarizing that many categorical differences were observed between the two cell populations in terms of significant up- and downregulation. The following specific gene targets have been labelled in the Vulcano plot: *Bmp2*, *Bmpr2*, *Il6*, *Id1*. In addition, the blue horizontal line shows the cut-off for the *P* value.

Statistical analyses of mRNA microarray expression revealed that 468 mRNAs are present at significantly different (p ≤ 0.05 and fold-change ≥10) levels in EC clones compared with control ECs (isolated from the CD117^−^ lung cell pool) (Fig. 4). Using Ingenuity Pathways Analysis software (Ingenuity Inc., Redwood City, CA), we found that these 468 differentially expressed mRNAs are over-representative of 63 canonical pathways. Significant differences in expression levels were found for genes with relevance to cell growth, apoptosis-regulation, endothelial cell biology/angiogenesis and PAH. For example, changes were detected for mRNAs related to endothelial cell biology/angiogenesis/vascular development, including upregulation of *Rab3b* (*P* = 0.00000035)^24^, uncoupling protein 2 (*Ucp2*) (*P* = 0.00000014)^25^, GATA protein 4 (*Gata4*) (*P* = 0.001), bone morphogenic protein 6 (*Bmp6*) (*P* = 0.001). Relative downregulation was for example observed for endothelial specific molecule 1 (*Esm1*) (*P* = 0.00000036) and apolipoprotein E (*Apoe*) (*P* = 0.004). Of particular relevance to the proliferative and anti-apoptotic phenotype of the EC clones were upregulation of genes associated with the clusters Cell Cycle:G2/M Damage Checkpoint Regulation (*P* = 0.00000262; z score = −1.89), Mitotic roles of Polo-like kinases (*P* = 0.000193; z score = 2.449) and cyclins and Cell cycle regulation (*P* = 0.000634; z score = 1.89). In addition, downregulation of genes was detected in the cluster Cell Cycle: G1/S Checkpoint Regulation (*P* = 0.0321; z score = −1). Figure 4 further shows the expression level of potential molecules of interest, such as *Bmp2*, *Bmpr2*, *Id1*, and *Il6*. A detailed identification of pathway/gene clusters is shown in Supplemental Table 1. Hence, the gene expression profile supports categorical differences in proliferative capacity and cellular signalling in CD117^+^ EC clones.

### Transplantation of EC clones promotes severe occlusive PH in rats

**Figure 5:**
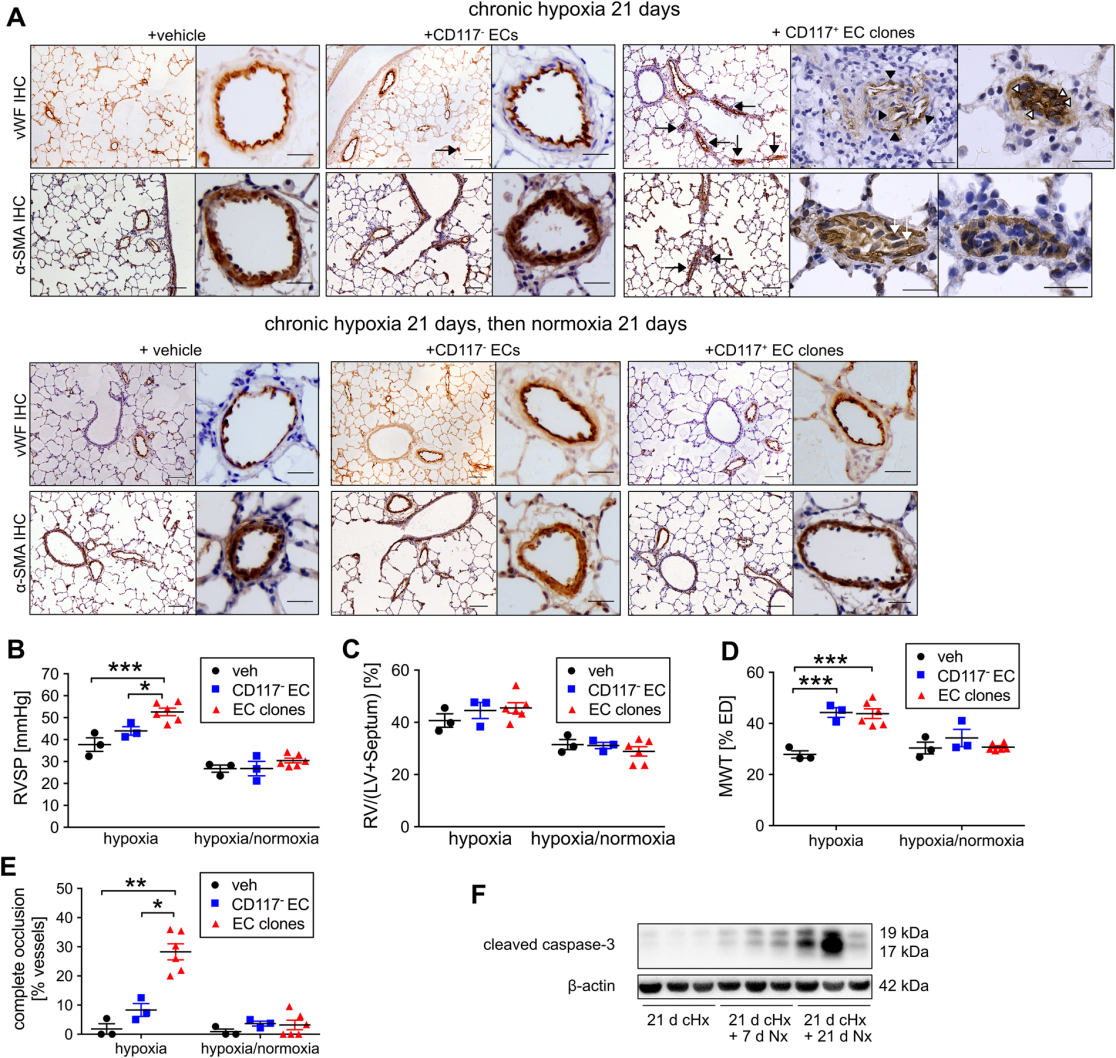
Transplantation of EC clones promotes reversible occlusive pulmonary arteriopathy and severe PH in rats exposed to chronic hypoxia. (**A**) Representative images of immunohistochemistry (IHC) for vWF and α-SMA (brown staining) demonstrating occlusive pulmonary artery remodelling in hypoxic rats that received EC clones (occlusion with vWF^+^ cells, arrows), but not in CD117^−^ EC transplanted rats (upper panel). Images show overview image obtained at 100× magnification and high-power images of representative pulmonary arteries (400× or 600× magnification). For the “chronic hypoxia 21 days + CD117^+^ EC clones” group, examples of occlusive vascular lesions are shown in high-power images at 600× magnification. In the low-power images, arrows indicate pulmonary arteries exhibiting occlusive lesions. In the high-power images, black arrowheads indicate multiple vascular channels within a pulmonary arterial lesion, and the white arrowheads indicate vWF^+^ cells occluding a pulmonary artery. Note the occlusion is cellular as demonstrated by haematoxylin^+^ cell nuclei (arrowheads). Some occluding cells were also α-SMA^+^(white arrows, lower panel). 21 days after cessation of cHx, the vascular changes were almost completely resolved (lower panel), except for elevated media wall thickness (MWT). Scale bars: 100 μm (low power), 25 μm (high power). (**B**) RVSP and (**C**) Fulton index. (**D**) MWT expressed as fraction of external diameter (ED). (**E**) Fraction of completely occluded pulmonary arteries. Scatter plots show single data points, mean and SEM. n = 3 (hypoxia, hypoxia+CD117^−^ EC), n = 6 (EC clones). *P < 0.05, ***P < 0.001. (**F**) Representative Western blot demonstrates time course of caspase 3 cleavage in the lung tissue of rats treated with EC clones, followed by 21 days of chronic hypoxia (cHx), 21 days of cHx and 7 days of normoxia (Nx), and 21 days of cHx and 21 days of Nx. The data indicate a progressive increase in the expression of cleaved caspase-3 in the lung tissue of EC clones + cHx rats after cessation of cHx. β-actin was used as loading control.

**Figure 6:**
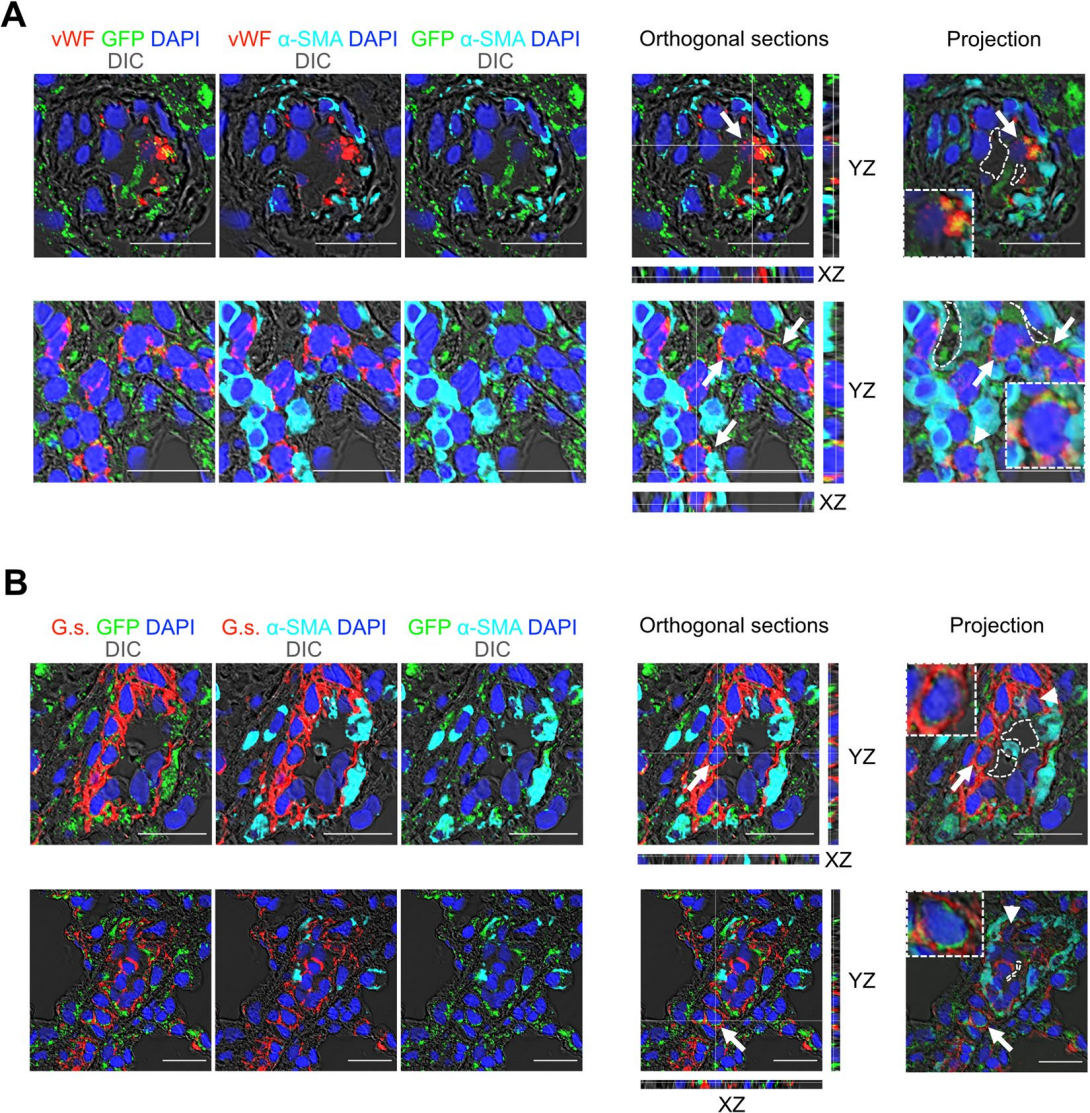
Confocal imaging of cell transplantation experiments. (**A**,**B**) Representative pseudo coloured optical sections obtained by confocal microscopy for immunofluorescence staining for GFP (green pseudocolour), α-SMA (cyan pseudocolour) and VWF (red pseudocolour) (**A**) and *Griffonia simplicifolia* lectin *(G*.*s*., red pseudolour), GFP (green pseudocolour) and α-SMA (cyan pseudocolour) (**B**) shows multiple vWF^+^ GFP^+^ cells (arrows) and VWF^+^ GFP^+^ αSMA^+^ cells (arrowhead), indicating EnMT, as well as *G*.*s*.^+^ GFP^+^ (arrows), *G*.*s*.^+^ GFP^+^ α-SMA^+^ (arrowheads) cells in and around the vascular lesions. GFP^+^ cells are derived from the transplanted EC clones at 21 days in hypoxia + EC clones. The findings of GFP^+^ cells in the lung vasculature 21 days after injection suggests engraftment of the EC clones. The images show the optical sections, orthogonal sections of Z-stacks and sum projection of the Z-stacks. Inserts in projection images show GFP^+^ EC or GFP^+^ EnMT cells in more detail. Nuclear staining with DAPI (blue pseudocolour), scale bar: 20 μm.

To identify the role of EC clones in the disease process of severe occlusive PH, we transplanted EC clones and control cells by intravenous injection *via* tail vein. Injection of EC clones in rats housed under conditions of normoxia did not result in changes in pulmonary arterial structure or increased right ventricular systolic pressure (RVSP) (Supplemental Fig. S6). Injection of EC clones followed by exposure to chronic hypoxia for 21 days resulted in occlusive pulmonary artery lesions and a significant increase in RVSP compared to hypoxia alone, hypoxia+vehicle and hypoxia+CD117^−^ control ECs (Fig. 5A,B). We did not find a significant increase in Fulton index with EC clone transplantation (Fig. 5C). During histological examination of the lungs, we found that media wall thickness (MWT), a marker of muscularization, was increased in the “hypoxia+EC clones” group (Fig. 5D). Furthermore, a substantial fraction of pulmonary arteries in “hypoxia+EC clones” rats were occluded by vWF^+^ cells (Fig. 5E). Some pulmonary arteries exhibited complex multicellular lesions, reminiscent of plexiform lesions in PAH patients. However, when rats were exposed to chronic hypoxia for 21 days after cell injection and then returned to normoxia for additional 21 days, the occlusive lesions disappeared, indicating reversibility in the absence of chronic hypoxia (Fig. 5A–E). We found increased levels of cleaved caspase-3 in the lung tissue of EC clone treated rats 7 and 21 days after cessation of chronic hypoxia, indicating that increased apoptosis may contribute to resolution of occlusive lesions during normoxia (Fig. 5F). In contrast to clonally expanded CD117^+^ ECs, CD117^+^ ECs without clonal expansion failed to generate hemodynamically relevant occlusive lung vascular lesions even with repeated injections (Supplemental Fig. S7). While we did not identify any EGFP^+^ cells in the RV of cHx+EC clones treated rats at day 21 (Supplemental Fig. S8), we found many cells of endothelial lineage in the vascular lesions with EGFP expression that were hence derived from the transplanted EC clones (Fig. 6). Using flow cytometry, we quantified the number of EGFP^+^ cells in one lobe of the right lung and found that 1.3 ± 0.34% (mean±SEM, n = 3) of cells in this lobe were GFP^+^ and hence derived from EC clones (representative dot plot in Supplemental Fig. S9). In addition, we also found evidence for GFP^+^ cells co-expressing endothelial and mesenchymal markers by identifying vWF^+^ α-SMA^+^ GFP^+^ and *Griffonia simplicifolia* lectin-binding α-SMA^+^ cells, indicating that some EC clones were undergoing endothelial-to-mesenchymal transition (EnMT) *in vivo* (Fig. 6).

### Gene expression profile in rat lungs after transplantation of EC clones

**Figure 7:**
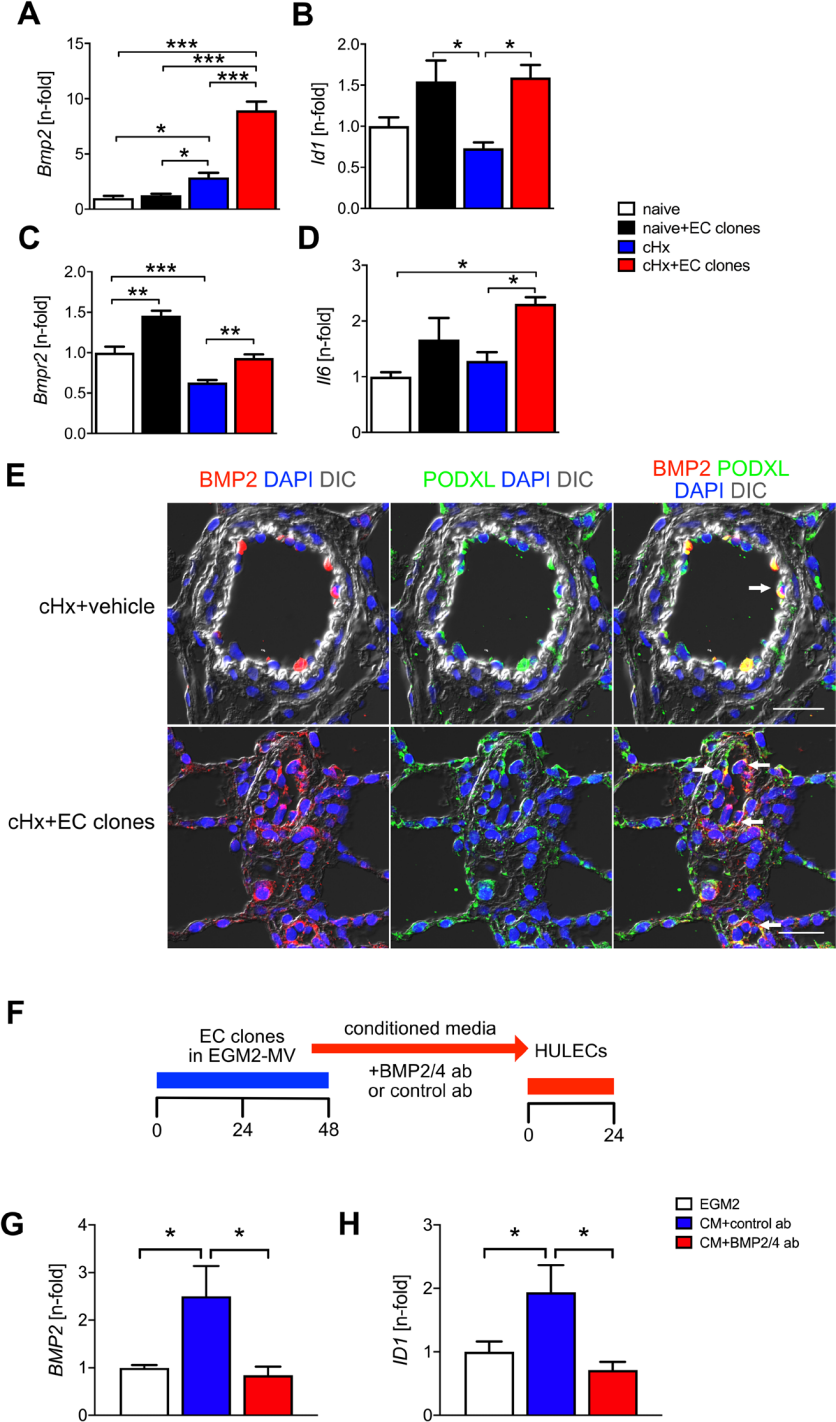
Lung tissue expression of genes with relevance to BMP pathway, inflammation and PAH pathogenesis after transplantation of EC clones. mRNA expression (qRT-PCR) of (**A**) *Bmp2*, (**B**) *Id1*, (**C**) *Bmpr2*, (**D**) *Il6* after EC clone transplantation compared with chronic hypoxia or normoxia and vehicle. n = 3 per group. (**E**) Localization of BMP2^+^ PODXL^+^ cells (BMP2^+^ ECs) in the pulmonary arteries of cHx+vehicle and cHx+EC clones rats. BMP2 is shown in red pseudocolour, and PODXL is shown in green pseudocolour. Whereas normal rat lung contains multiple BMP2^+^ ECs (one representative cell is indicated by an arrow) and multiple BMP2^−^ ECs, accumulation of BMP2^+^ ECs is vastly increased in cHx + EC clones rats, and multiple BMP2^+^ ECs are found in occlusive pulmonary arterial lesions (arrows). This staining includes no GFP stained cells and only demonstrates the increase in BMP2^+^ ECs after EC clone transplantation. Scale bar: 25 μm. Nuclear staining with DAPI (blue pseudocolour). (**F**) Diagram of the experimental approach for *in vitro* experiments. First, rat CD117^+^ EC clones were cultured for 48 h in EGM-2MV medium. Then, this conditioned media (CM) was transferred after addition of anti-BMP2/4 antibody (ab) or control ab to HULECs. After 24 h, HULECs were removed for gene expression analysis. (**G,H**) mRNA expression of *BMP2* (**G**), and *ID1* (**H**) in HULECs after 24 h exposure to media conditioned for 48 h by rat lung CD117^+^ EC clones. n = 9–12 per group. *P < 0.05, **P < 0.01, ***P < 0.001.

To identify whether transplantation of EC clones causes changes in the gene expression profile of rat lungs, we performed qRT-PCR. We found that transplantation of EC clones promoted the increase of angiogenic BMP2 in hypoxic rats. The ‘hypoxia+EC clones’ group showed increased expression of *Bmpr2* and *Id1*, a downstream target of the BMP/Smad1 pathway (Fig. 7). On the other hand, *Il6*, *a* gene with potential relevance in PAH pathobiology^26,27^, was increased in the ‘hypoxia+EC clones’ group (Fig. 7). While transplantation of CD117^−^ ECs combined with exposure to cHx promoted *Bmp2* expression, expression of *Bmpr2* and *Il6* were reduced compared to cHx alone (Supplemental Fig. S10). Using double immunofluorescence, BMP2 expression was localized to a subset of ECs in alveolar capillaries and pulmonary arteries in rats exposed to cHx alone (Fig. 7E). In the cHx + EC clones group, multiple ECs with high BMP2 expression were found to occlude the pulmonary arteries, forming occlusive lesions. To determine whether expression of BMP ligands in EC clones contributes in a paracrine manner to increased BMP ligand expression and proliferation in lung resident ECs, we transferred conditioned media from EC clones to immortalized human lung ECs (HULECs) while blocking BMP2 and BMP4 using a neutralizing antibody. mRNA expression of *BMP2* and the BMP downstream target *ID1* was increased in the conditioned media+control antibody group, and these increases were abolished by BMP2/4 inhibition (Fig. 7F–H). Hence, we identified increased expression of BMP2 in ECs, likely as a consequence of the transplanted EC clones.

### *In vitro* exposure to hypoxia decreased BMP pathway activity in CD117^+^ EC clones

Because hypoxia has been identified to alter expression of genes with relevance in PAH, we tested whether exposure to hypoxia alters gene expression and BMP pathway activity in CD117^+^ EC clones. We found that exposure to 48 h of hypoxia caused downregulation of *Bmp2* and *Bmpr2*, as well as reduced Smad1/5/9 phosphorylation (Supplemental Fig. S11).

## Discussion

Over the past decades, changes in endothelial function and phenotype have been described in the vascular lesions of PAH patients. These range from increased susceptibility to apoptosis, aberrant or impaired angiogenesis and unchecked proliferation to EnMT^3,21,28^. One concept suggests that the aberrant ECs in plexiform lesions of IPAH patients develop by clonal selection of primitive endothelial-like stem cells, potentially in response to widespread endothelial apoptosis^4^. Primitive endothelial precursors are a common source of hematopoietic precursors, mesenchymal precursors and definitive ECs during vascular development, and the adult human pulmonary vasculature harbours endothelial precursor cells with high proliferative capacity^11,14,15,17,29,30^. These cells could serve as source for aberrant angiogenesis and mesenchymal cells in PAH. Recent publications indicate that CD117, the ligand for SCF, is expressed in pulmonary endothelial precursors with high proliferative capacity, and CD117^+^ cells accumulate in pulmonary vascular lesions of PAH patients^5,15,17,31^.

We hypothesized that clonal selection of CD117^+^ ECs yields primitive EC clones that exhibit extensive proliferation, angiogenic sprouting, and the ability to give rise to mesenchymal cells and definitive endothelium. We further hypothesize that transplantation of these clonally selected primitive ECs promotes occlusive pulmonary arteriopathy and PH.

We first expanded on the work by Montani *et al*. on CD117^+^ cells in PAH^5^. While Montani *et al*. showed that CD117^+^ cells in blood and lungs from PAH patients are at least in part mast cells, we demonstrate here that in PAH lung vascular lesions a relevant portion of the CD117^+^ cells express endothelial markers, providing a rationale to further investigate CD117^+^ ECs in PAH.

Using an *in vitro* four generation clonal expansion protocol, we successfully enriched primitive clonal ECs from CD117^+^ rat lung ECs. These cells expressed EC markers and showed typical endothelial function, including 2D and 3D formation of angiogenic tubes^14,15,32,33^. The ability for clonal expansion has been shown to be a marker of endothelial precursors or stem cells^17,34,35^. The CD117^+^ EC clones further gave rise to blood vessels in matrigel plugs *in vivo* in the study by Fang *et al*.^17^. It is important to note that clonal expansion is not an exclusive feature of CD117^+^ ECs, but can also be found in lung ECs in general^14,15^. Yet our data clearly show that clonal selection yields ECs with higher proliferative capacity, and our findings support previous concepts of selection pressure and clonal expansion as processes that yield the apoptosis-resistant, hyperproliferative ECs found in PAH^2^. Despite the importance of clonal selection, there is also clear evidence to support a role for SCF/CD117 signalling in PAH: First, CD117^+^ cells have been found in occlusive pulmonary arterial lesion in both human PAH and animal models of pulmonary hypertension^5,20,36^. Our current histological data expand on these publications by showing the presence of CD117^+^ ECs in occlusive lung vascular lesions using three different EC markers. Second, Fang *et al*. demonstrate in their publication that CD117-deficient ECs from mouse lungs are impaired in de-novo blood vessel formation, proliferation and colony formation in comparison to CD117-competent mouse lung^17^. And third, our data show that blocking SCF/CD117 signalling reduces angiogenesis and cell viability in rat lung CD117^+^ EC clones.

In addition to the angiogenic and proliferative phenotype of the CD117^+^ EC clones, we also observed phenotypic plasticity of these cells towards smooth muscle-like cells as part of their stem-like features, as culture in MEM or SmGM2 was sufficient to cause EnMT. It is possible that the previous clonal expansions promoted an EnMT-prone phenotype, as clonal expansion has been previously associated with partial EnMT^18^. Further, osteogenic differentiation was readily induced using appropriate culture conditions, similar to bone marrow-derived mesenchymal stem cells. While our cells retained in part endothelial markers, our data also show that CD117^+^ EC clones switch phenotype when cultured in a basal medium (MEM) instead of EGM2 and do not require additional stimulation with TGF-β. Yet additional stimulation with TGF-β1 greatly enhanced mesenchymal marker expression, consistent with previous studies^37,38^. In contrast, combination of TGF-β1 and PDGF-B led to more proliferative gene expression pattern consistent with PDGF-B-mediated effects enhancing proliferation^39^. Using another non-endothelial growth medium, SmGM2, we found that EnMT induced by growth medium variation is prevented by chemical inhibition of the TGF-β type I receptor ALK5. Our data further demonstrate that the balance between Smad2-dependent TGF-β and Smad1/5/9-dependent BMP signalling is a strong regulator of the phenotypic switch in CD117^+^ EC clones, because ALK5 inhibition not only reduces TGF-β signalling, but also enhances BMP signalling and restores BMPR2 expression. This is consistent with a recent publication showing that dysfunctional BMPR2 signaling promotes the transition of PAH ECs to smooth muscle-like cells^13^. In addition to priming towards EnMT by clonal expansion, these results could also point towards a substantial plasticity of the CD117^+^ EC clones not only towards the endothelial lineage, but also towards mesenchymal lineages, more akin to the primitive ECs that give rise to hematopoietic, endothelial and mesenchymal lineages during cardiovascular development^10,11^. CD117 is expressed during the endothelial lineage specification from mesoderm, further supporting our concept^40^. In agreement with the available literature, we were able to promote angiogenic function of the EC clones by inhibition of ALK5-dependent TGF-β signaling^41,42^.

To further identify whether primitive, clonally selected CD117^+^ ECs indeed contribute to the occlusive arteriopathy typical of PAH, we took a two-step approach with initial clonal selection *in vitro* followed by injection into living animals. Our approach is relevant to the development of occlusive pulmonary arteriopathy in PAH, because we were able to reproduce occlusive arteriopathy by injection of hyperproliferative ECs, which have been selected *in vitro* by repeated clonal expansion. Clonal selection has been described as a potential mechanism leading to EC proliferation in plexiform lesions in PAH^4^. Our approach bypasses the need for inducing clonal selection via apoptosis using compounds such as SU5416, which can have enduring effects on cell function, including induction of senescence^43^. Exposure to chronic hypoxia for 21 days resulted in occlusive pulmonary arteriopathy and increased RVSP. The role of hypoxia for promoting proliferation of endothelial precursor cells has been shown previously in the lung^44^. Our results support a concept that the aberrant ECs in PAH occlusive vascular lesions develop by clonal selection from endothelial-like stem cells^4^. In addition, our results also demonstrate that the transplanted cells exhibited a microvascular endothelial phenotype and underwent EnMT. In our experiments, occlusive arteriopathy was indeed induced by transplantation of CD117^+^ EC clones, but these changes were reversible after cessation of chronic hypoxia. While these results clearly demonstrate that transplantation of highly proliferative, clonally expanded ECs promotes occlusive arteriopathy in the context of chronic hypoxia, our data also indicate that highly proliferative ECs alone may not be sufficient to promote persistent or progressive occlusive arteriopathy without hypoxia. Our data further show that the resolution of the occlusive lesions is associated with increasing degree of apoptosis in the lungs, suggesting that hypoxia may drive proliferation and survival^44^. There are multiple reasons why CD117^+^ EC clones failed to maintain progressive angio-occlusive PH after cessation of hypoxia. For example, previous work by Diebold *et al*. has demonstrated that loss of functional BMPR2 expression results in persistent vascular changes and PH in mice exposed to chronic hypoxia^28^, and our results clearly indicate that BMPR2 and BMP2 expression was increased after transplantation of CD117^+^ EC clones, likely as a consequence of  increased production of BMP ligands by the clones. Our data further show that CD117^+^ EC clones develop impaired BMPR2 signalling after exposure to hypoxia *in vitro*, indicating that hypoxia promotes a PAH phenotype that may be reversible after cessation of chronic hypoxia. Hence, additional functional, genetic and epigenetic changes may be necessary to generate a dysfunctional endothelial phenotype that shows elements of increased inflammatory response, an apoptosis-prone fraction, and unchecked proliferation in surviving cells^3,28^. Our data however support the presence of EnMT *in vivo* as another potential mechanism of occlusive pulmonary vascular remodelling^13,21^. In addition, as endothelial apoptosis has been demonstrated as one initiating force of lung vascular remodelling in various PH models, the extent of vascular and in particular endothelial injury may not be sufficient in the initial phase for persistent PH in our model^45–47^. This is consistent with a concept that vascular rarefaction is important to establish progressive PH in addition to the proliferative remodelling of pulmonary arteries^48,49^.

Several limitations of the study should be noted, however. First, while we observed that after injection of EC clones there was increased RVSP after exposure to cHx, there was no increase in right ventricular hypertrophy. We did not further investigate the detailed mechanism of a potential right ventricular maladaptation after injection of EC clones. However, our finding of increased pulmonary expression of IL-6 in the cHx + EC clones group provides one potential explanation for right ventricular maladaptation as increased IL-6 levels have been associated with impaired right ventricular adaption in two studies, including the Multi-Ethnic Study of Atherosclerosis (MESA)-RV study^50,51^. Second, the tail vein injection of *in vitro* clonally expanded ECs is an artificial means to test our hypothesis that clonally selected CD117^+^ ECs contribute to the occlusive pulmonary arteriopathy in PAH. We further acknowledge that this approach may not fully recapitulate all aspects of PAH pathophysiology, because unspecific entrapment of the highly proliferative ECs after injection may contribute to the resulting vascular phenotype.

In conclusion, we demonstrate the isolation and clonal expansion of primitive CD117^+^ ECs with bipotent lineage differentiation potential into endothelial and mesenchymal lineages. Whereas transplantation of these cells induced occlusive arteriopathy and increased PH in rats exposed to chronic hypoxia, occlusive arteriopathy was largely resolved after cessation of hypoxia. These data indicate a contribution of CD117^+^ primitive ECs to the occlusive pulmonary arteriopathy in PAH, but also demonstrate that additional mechanisms are likely required.

## Methods

### Human tissue samples

All experiments were performed according to Paraffin-embedded, formalin-fixed human lung tissue samples were obtained as de-identified specimen from the Department of Pathology, University of Denver at Colorado, and the Pulmonary Hypertension Breakthrough Initiative (PHBI) under protocols approved by the Pulmonary Hypertension Breakthrough Initiative, and the University of Pennsylvania and University of Colorado institutional review boards in conformity with ethical guidelines of the Declaration of Helsinki of 1975, as revised in 1983. Informed consent was waived by these institutional review boards. The use of these de-identified human lung tissue sections was deemed as “non-human subjects research” by the office of research subjects protection at Virginia Commonwealth University and the Ohio State University.

### Animal housing

Male SD-Tg(UBC-EGFP)2BalRrrrc rats (Rat Resource & Research Center, University of Missouri, Columbia, MO) with ubiquitous heterozygous expression of enhanced green fluorescent protein (EGFP) (EGFP^+^ rats) and male Sprague-Dawley rats (Envigo Laboratories, Indianapolis, IN) were housed at 12 h/12 h light/dark cycle with food provided ad libitum. All procedures were approved by the Institutional Animal Care and Utilization Committee at VCU and OSU, and performed in accordance with the Guide for the Care and Use of Laboratory Animals (8th edition).

### Isolation and clonal expansion of CD117^+^ ECs

Isolation of CD117^+^ ECs has been previously described^45^. In short, after thorough rinsing with PBS (60–80 ml) via a right ventricular catheter to remove circulating hematopoietic cells, rat lungs were removed from euthanized EGFP^+^ rats and a single cell suspension was prepared from the peripheral lung tissue (hilar regions excised) using a modified protocol according to van Beijnum *et al*.^52^. In brief, tissue was minced into <1 mm^3^ pieces, and digested in 0.1% collagenase II/2.5 U/ml dispase solution (Thermo Fisher Scientific, Waltham, MA) for 30 min at 37 °C. Then, 0.1% DNase was added to the solution (Sigma-Aldrich, St. Louis, MO) and the tissue pieces were incubated for another 30 minutes at 37 °C. CD117^+^ and CD117^−^ cells were obtained by immunomagnetic sorting using magnets and the “Any Species positive selection” kit from Stem Cell Technologies (Vancouver, BC) using CD117 antibody (bs-0672R, Bioss, Woburn, MA). Then, hematopoietic cells were removed from CD117^+^ and CD117^−^ cell fractions by negative sorting with bead-conjugated antibodies directed against CD5, CD11b/c and CD45 (pan-CD45) using the Pluribeads technology (Pluribeads, Leipzig, Germany). A mix of the following antibodies was used for negative selection: CD5 (sc-53054, Santa Cruz Biotechnology, Santa Cruz, CA), CD45 (554875, BD Biosciences, Franklin Lakes, NJ) and CD11b/c (554859, BD). To enrich the endothelial fraction, cells were enriched for CD31 using a CD31 antibody (555025, BD) coupled with pluribeads. The hereby obtained CD117^+^ lin^−^ CD31^+^ cells (CD117^+^ ECs) and CD117^−^ lin^−^ CD31^+^ cells (CD117^−^ ECs, control cells) were cultured on type I collagen coated dishes with microvascular endothelial growth medium (EGM-2MV, Lonza, Walkersville, MD or EGM-MV2, Promocell, Heidelberg, Germany).

For clonal expansion passage 1 CD117^+^ ECs were seeded at limiting dilution (1 cell per well) in 96 well plates. The presence of 1 cell per each well was confirmed by microscopy. After 14 days, the largest colonies with typical endothelial “cobblestone” morphology were identified and the 6 largest endothelial colonies were used for the next round of clonal expansion. This procedure was repeated until fourth generation clonal colonies were obtained. Selection of the largest clonal colonies ensured selection of the clones with the highest proliferation and self-renewal capacity. For experiments, the cells were used after 4 clonal expansions and in the first 5 post-clonal passages.

### Flow cytometry

EC clones and CD117^−^ control ECs were stained with fluorescent label-conjugated antibodies against CD144 (bs-0878R, Bioss, Woburn, MA), VEGFR2 (bs-05665R, Bioss), CD105 (bs-4909M, Bioss), CD34 (sc-7324, Santa Cruz Biotechnology, Santa Cruz, CA), CD117 (bs-0672R, Bioss), CD45 (202212, Biolegend, San Diego, CA), CD11b/c (554862, BD, Franklin Lakes, NJ) and CD133 (bs-0209R, Bioss) according to previously published standard protocols^53^. For staining of intracellular vWF (ab8822, Abcam, Cambridge, MA), permeabilization of cells was performed using Cytofix/Cytoperm reagent (BD) according to manufacturer’s recommendation. Isotype control antibodies derived from same species and conjugated to the respective fluorochrome were used as controls. Flow cytometry data was obtained with a BD FacsCanto (BD) and analyzed using FlowJo 10 (FlowJo, Ashland, OR).

### Isolation and characterization of bone marrow mesenchymal stem cells (BM-MSCs)

See supplemental methods.

### Mesenchymal lineage differentiation assays

See supplemental methods.

### Endothelial function assays

See supplemental methods.

### EC transplantation procedure in rats

1 × 10^6^ EC clones, control cells (ECs derived from CD117^−^ cell fraction) or vehicle (PBS) were injected intravenously by tail vein. The animals were randomly assigned to the different categories at the beginning of the experiments. Then, rats were either housed at room air (normoxia, Nx) or chronic hypoxia for 7 or 21 days (Hx, inspiratory O_2_ fraction 10%). At days 21, 28 and 42, the animals underwent right heart catheterization to obtain right ventricular systolic pressure under Ketamine/Xylazine anaesthesia as published previously^20,53^. Animal treatments and hemodynamic measurements were performed by a member of the research staff who was unaware of the treatment groups. After hemodynamic evaluation, the rats were euthanized by abdominal bleeding under anaesthesia in accordance with the guidelines of the Panel on Euthanasia of the American Veterinary Medical Association. Lung and heart were removed *en bloc*. The left lung was inflated with low-melting agarose (0.5%) at a pressure of 20 cmH_2_O and fixed in 10% neutral buffered formalin, then processed and embedded in paraffin for histology. The tissue of the right lung was divided: From one lobe, a single cell suspension was prepared for flow cytometry as previously described^45,53^. The remaining lobes were snap frozen for molecular biology. For repeated injection of CD117^+^ ECs, 1 × 10^6^ ECs were injected intravenously by tail vein once a week for three weeks, while animals were exposed to normoxia (room air) or chronic hypoxia. At day 21, animals underwent hemodynamic evaluation and tissue harvest as described above.

### Histology

See supplemental methods.

### Confocal microscopy

Confocal microscopy was performed with upright Zeiss LSM700 or inverted Zeiss LSM710 laser scanning confocal microscopy systems located at the VCU Department of Anatomy and Neurobiology Microscope Facility. Additional confocal imaging was obtained with an inverted Olympus FV3000 confocal microscope system located at the OSU Campus Microscopy and Imaging Facility (CMIF). The images were assembled with Fiji software.

### RNA isolation, quantitative real-time PCR (qRT-PCR), protein isolation, western blot

See supplemental methods.

### Affymetrix mRNA expression profiling

100 ng of total RNA was processed with the Affymetrix GeneChip™ WT PLUS Reagent Kit (Thermo Fisher Scientific, Waltham, MA) according to the manufacturer’s recommendations. The resultant labelled RNA was hybridized to Affymetrix Clariom™ S Rat microarrays for 16 hr and washed and stained on the Affymetrix GeneChip™ Fluidics Station 450 using the FS450_0007 fluidics script and then scanned on the Affymetrix GeneChip™ Scanner 3000. Resulting data were normalized in Affymetrix Expression Console software using gene-level SST-RMA and exported into Partek Genomics Suite version 6.16.0308 for analyses.

One-factor ANOVA was used to compare mRNA expression differences between EC clones and control EC cell lines, using FDR (step up) p < 0.05 and fold change >2 as filters. Ingenuity Pathway Analysis software (Qiagen, Germantown, MD) was used to analyse the biological relevance and predicted pathways based on genes that significantly differed between EC clones and control ECs. Predicted changes in gene activity were expressed as z score with positive z score indicating increased activation and negative z score indicating decreased activation. The stringent cut-off values of p ≤ 0.05 and a fold-change (FC) cut-off of ±10 were applied to this analysis.

### Quantification of cell engraftment by flow cytometry

Single cell suspensions from one lobe of the right lung were stained with fluorescent label-conjugated antibodies against GFP (sc-8334, Santa Cruz Biotechnology) according to previously published standard protocols^53^. The fraction of cells expressed the various markers was obtained for each marker by flow cytometry with a BD FacsCanto (BD Biosciences, San Jose, CA) and analysis in FlowJo (FlowJo, Ashland, OR).

### Statistical analysis

Data are presented as mean ± SEM. The groups were compared in normally distributed data sets with 2-tailed unpaired Student’s *t* test, 1-way or 2-way ANOVA followed by Holm-Sidak multiple comparison test. Nonparametric statistical testing was done with 2-tailed Mann-Whitney or Kruskal-Wallis test with two-stage linear step-up procedure of Benjamini, Krieger and Yekutieli as post-hoc comparison test. Statistics and graphs were done with GraphPad Prism 7.0 (GraphPad Software). *P* < 0.05 was considered significant.

## Supplementary information

Supplementary Information.

Table S1.

## Data Availability

The datasets generated and/or analyzed during the current study are available from the corresponding author on reasonable request.
