# Supplementary material for: Clonally selected primitive endothelial cells promote occlusive pulmonary arteriopathy and severe pulmonary hypertension in rats exposed to chronic hypoxia
Source: Sci Rep. 2020 Jan 24;10:1136. doi: 10.1038/s41598-020-58083-7 (PMC6981224; doi:10.1038/s41598-020-58083-7)
Supplement: Supplementary file 1 — Supplementary Information. [file 41598_2020_58083_MOESM1_ESM.pdf]

SUPPLEMENTAL METHODS AND DATA

Clonally selected primitive endothelial cells promote occlusive pulmonary arteriopathy and severe pulmonary hypertension in rats exposed to chronic hypoxia

Authors:
Aneel R. Bhagwani [1, 2&], Daniela Farkas [1&], Brennan Harmon [3], Kayla J. Authelet [3], Carlyne D. Cool [4], Martin R.J. Kolb [5], Elena A. Goncharova [6], Mervin C. Yoder [7], Matthias Clauss [8], Robert Freishtat [3], Laszlo Farkas [1,2*]

[&] Equally contributing authors
* Corresponding author

SUPPLEMENTAL METHODS

Isolation and characterization of bone marrow mesenchymal stem cells (BM-MSCs)

Bone marrow cells were obtained from heterozygous SD-Tg(UBC-EGFP)2BalRrrrc rats (Rat Resource & Research Center, University of Missouri, Columbia, MO). These rats ubiquitously express enhanced green fluorescent protein (EGFP) under control of a ubiquitin promoter. Adult rats were euthanized by exsanguination under anaesthesia with Ketamine/Xylazine, and femora, tibiae and humeri were removed, cleaned and kept in ice-cold MEM (Gibco, Life Technologies) for less than 2 h until use. Bones were cut open on both ends and flushed with ice-cold MEM using an 18-gauge needle into a 10cm culture dish. After straining through a 70µm filter, gradient centrifugation in Histopaque at 1:3 volume ratio of BM solution/Histopaque was performed. Mononuclear cells were removed and washed twice with MEM medium. Then, cells were plated in complete growth medium (MEM supplemented with 10% FBS, 100 U/ml of penicillin, 100 µg/ml streptomycin and 2mM L-Glutamine [all from Gibco]) in a 37˚C 5% CO_2 incubator at 100% humidity. After 5 days in culture, non-adherent

cells were removed by adding fresh MEM and only MSCs remained in culture dishes due to plastic adherence. MEM was changed every three to four days, until 70-80% confluence was reached. MSCs were used in passage two to four for experiments. For flow cytometry, 1×10^6 cells were diluted in 100μl of 1% FBS/PBS. Cells were stained using directly labelled antibodies against CD45 (PE-Cy5, 559135, BD Pharmingen, Franklin Lakes, NJ), CD34 (PE-Cy7, sc-7324, Santa Cruz Biotechnology, Santa Cruz, CA), CD73 (custom labelling of 551123 with APC, BD Pharmingen) and Thy1 (APC-Cy7, 561401, BD Pharmingen) or corresponding isotype controls for 30 minutes on ice. After the staining was finished, cells were fixed in 1% paraformaldehyde for flow cytometry analysis with FACSCanto II and FlowJo 10 Software.

Phenotypic switch experiments with media, recombinant growth factors and ALK5 inhibition

To identify whether the culture medium determines the phenotype of $CD117^+$ EC clones, we cultured EC clones for 7 days in minimal essential medium with 10% FBS and 100 IU/ml Penicillin and 100 μg/ml Streptomycin. In addition, cells were stimulated in MEM with either 5 ng/ml recombinant human transforming growth factor-β1 (TGF-β1) or a combination of 5 ng/ml TGF-β1 and 25 ng/ml platelet-derived growth factor-B (PDGF-B). In additional experiments, EC clones were cultured in smooth muscle cell growth medium (SmGM2) for 3 days with vehicle or with the ALK5 inhibitor SB-431542 (10 μM).

Inhibition of stem cell factor (SCF)

To determine the role of SCF/CD117 signaling in angiogenic phenotype of $CD117^+$ EC clones, we performed matrigel 2D angiogenesis assays as described below under endothelial

function assays under treatment with 10 ng/ml anti-SCF neutralizing antibody (R&D Systems, Minneapolis, MN, AB-255-NA) or unspecific goat serum (=control antibody, 5 ng/ml).

Immortalized human lung microvascular ECs

Human lung microvascular ECs immortalized using pSVT vector, a pBR-322 based plasmid containing the coding region for Simian virus 40A gene product, large T antigen, were obtained from the American Type Tissue Collection (ATCC, Manassas, VA). These immortalized ECs (HULEC-5a, CRL-3244, called HULECs in the manuscript) were cultured in EGM-2MV media.

Conditioned media experiments with inhibition of BMP2 and 4.

To identify whether BMP ligands released from rat lung CD117$^+$ EC clones promote expression of BMP2/4 and BMP receptor 2, and promotes proliferation in resident lung ECs, CD117$^+$ EC clones conditioned complete EGM-2MV media for 48h, which was then transferred to HULECs for 24h. Prior to incubation of conditioned media with HULECs, the conditioned media was treated with 15 µg/ml anti-BMP2/4 antibody (R&D Systems MAB3552) or isotype control antibody (R&D Systems MAB002). At the end of the experiments, cells were removed for RNA isolation and qRT-PCR.

Exposure to hypoxia

To identify the effect of exposure to hypoxia, we kept CD117$^+$EC clones in 5% O_2 for 48 hours. Hypoxia was induced using a modular incubator chamber with O_2 fraction adjusted to 5% by using a 95% N_2/ 5% O_2 gas mixture as described previously by Wu and Yotnda [1]. Cells cultured in normoxia were used as controls.

Cell viability

Cell viability was tested using CellTiter-Glo (Promega, Madison, WI) according to the manufacturer's instructions. Read-outs were performed with GloMax Luminometer (Promega).

Mesenchymal lineage differentiation assays

Adipogenic differentiation of MSCs and EC clones was performed by plating 10^4 cells/cm^2 on 2-well Nunc Lab-Tek chamber slides and the cells were pre-cultured with compete growth medium for 3 days before inducing adipogenesis using StemPro® Adipogenesis Differentiation Kit (Gibco) according to the manufacturer's instructions. The adipogenesis differentiation medium was changed every 3 days. 28 days after induction of adipogenesis, cells were stained with Oil Red O to visualize lipid droplets and counterstained with Harris' Hematoxylin. For osteogenic differentiation of BM-MSCs and EC clones, 5×10^3 cells/cm^2 were seeded on 2-well chamber slides and cultured with complete growth medium for 3 days before inducing osteogenesis using the StemPro Osteogenesis Differentiation Kit (Gibco) according to the manufacturer's instructions. The osteogenic differentiation medium was changed every 3 days. 28 days after induction of osteogenesis, cells were washed with Dulbecco's PBS and fixed with 4% paraformaldehyde for 30 min. Cells were stained with 2% Alizarin Red S solution (pH 4.2) for three min and washed with distilled water. Cells were examined for lipid droplets or calcium deposition with AXIO imager.A1 microscope, Axiocam HRc camera and Axiovision software (all Zeiss). Negative controls without induction of adipogenesis and osteogenesis in BM-MSCs were run in parallel.

Endothelial function assays

Matrigel tube formation assay: 5×10^4 EC clones were plated per well in EGM-2MV on matrigel (BD Biosciences) on Ibidi angiogenesis µ slides (Ibidi, Fitchburg, WI). Images were taken with an inverted IX70 microscope and XM10 camera (Olympus, Waltham, MA). Additional treatments were SB421543 (10 µM) and vehicle dissolved in EGM-2MV. For quantification, complete wells were acquired using a 4× objective with the image stitching function of cellSens Dimension software (Olympus). Total tube length was measured using Angiotool (National Cancer Institute) [2].

Binding of *Griffonia simplicifolia* (GS) lectin: Fluorescent-labelled GS lectin (Vector laboratories, Burlingame, CA) was incubated with EC clones and control ECs for 2 hours, followed by counterstaining with DAPI for 5 min. After mounting with Slowfade Gold (Invitrogen), GS labelling was identified using an LSM 700 confocal microscope (Zeiss).

Fibrin sandwich angiogenesis assay: The Fibrin angiogenesis assay kit was used as per manufacturer's recommendations for the 3D angiogenesis sandwich assay (Merck Millipore, Burlington, MA). In short, 30 µl fibrinogen was layered in each well of a 96 well plate, and 20 µl of thrombin were added in each well. Fibrinogen/thrombin was polymerized for 20 min at 37˚C. Then, 5.4×10^3 cells were layered over fibrin in 100 µl EGM. After incubation overnight at 37˚C, culture media was removed and another layer of 30 µl fibrinogen/20 µl thrombin was added and polymerized. After 48h, cells were permeabilized with 0.1% Triton-X, stained with Rhodamine-labelled phalloidin and DAPI for imaging. Z-stacks were acquired with an inverted Zeiss LSM710 laser scanning confocal microscopy systems located at the VCU Department of Anatomy and Neurobiology Microscope Facility. Intensity projections of Z stacks were generated with Fiji software [3].

Spheroid tube sprouting assay: To confirm the vasculogenic and angiogenic properties of EC clones in 3D, the cells were seeded in low attachment 24 well plates (NUNC, ThermoFisher, Waltham, MA and Corning, Corning, NY) to evaluate formation of spheroids without adhesion of cells to extracellular matrix as described previously by Heiss *et al.* [4]. After 24h, growth medium was removed, and the spheroids or cell structures were covered with a 1:1 volume mixture of matrigel and EGM-2MV growth media. Additional treatments were SB421543 (10 µM) and vehicle in EGM-2MV. Outgrowth of angiogenic tubes was documented after 7 days and 14 days with an IX70 microscope and XM10 camera with cellSens Dimension software (Olympus). Sprouting area was measured in Fiji.

Matrigel plug assay: To confirm vasculogenic/angiogenic function of EC clones *in vivo*, a matrigel plug assay was performed as previously described by Alvarez *et al.* [5]. In short, 375,000 cells derived from each EC clone were suspended in 750 µl of a 2:1 Matrigel:EGM mixture, which was injected into the right and left flank of naive Sprague Dawley rats. After 14 days, Matrigel plugs were harvested from the anesthetized animal and fixed in formalin, processed, embedded and stained for GFP/counterstained with DAPI. Images were acquired by confocal microscopy.

Histology

Histological stainings were performed as published previously on 3-5 µm sections of paraffin-embedded, formalin-fixed lungs [6–9]. IHC and double/triple IF stainings were performed as previously published [6–9]. The following primary antibodies were used: α-SMA (M085129-2, DAKO Agilent, Santa Clara, CA), BMP2 (Abcam, Cambridge , MA, ab6285), CD117 (AF1356, R&D Systems, Minneapolis, MN and LS-C78828, LifeSpan Biosciences, Seattle, Washington), CD31 (NBP1-49805, Novus Biologicals, Centennial, CO), GFP (sc-

8334, Santa Cruz Biotechnology, Santa Cruz, CA), GFP (Cell Signaling, Danvers, MA, #2555), PODXL (Bioss, Woburn, MA, bs-1345R), vWF (A008229-5, DAKO Agilent, Santa Clara, CA), vWF (MAB-3442, Millipore Sigma, Danvers, MA). In addition, incubation with rhodamine-labelled *Griffonia simplicifolia* lectin (Vector Laboratories, Burlingame, CA, RL-1102) was used as additional marker of microvascular ECs.

For measurement of muscularization in pulmonary arteries, images of α-smooth muscle actin (α-SMA)-stained sections were randomly taken with an AXIO imager.A1 microscope, Axiocam HRc camera and Axiovision software (all Zeiss, Jena, Germany) at 100× magnification. Objectivity was ensured by providing each animal with a numerical code to mask treatment groups. Media wall thickness (MWT) was calculated from media thickness (MT) and external diameter (ED) as published before [8,9]. Pulmonary arteries were categorized as follows: small-sized 25 μm < ED < 50 μm, medium-sized 50 μm ED < 100 μm. For each animal, 30-40 pulmonary arteries were measured in two orthogonal directions using Fiji software.

Vascular occlusion was quantified in sections stained for vWF IHC as previously published by us following the classification initially described by Oka et al. [6,9,10]. A blood vessel was categorized as completely occluded, when >75% of the luminal area was occluded by vWF^+ cells.

To quantify IHC, the number of positive cells per pulmonary artery was counted as previously described in a blinded and randomized manner [6–9].

To quantify $CD117^+$ cells in fluorescence stainings of human patients and control subjects, the number of $CD117^+$ cells and total cells was counted per pulmonary artery using Fiji software and analysed as previously published [11]. The characterization of the studied

patients and controls has been published in [11]. The samples used in this study were serial sections of the ones used in [11].

RNA isolation and quantitative real-time PCR (qRT-PCR)

mRNA extraction was done with the miRNeasy Mini Kit (Qiagen, Valencia, CA) according to manufacturer’s instructions. Reverse transcriptase reaction was done according to established standard protocols. In brief, DNAase I treatment was followed by transcription of 1 µg of RNA with random hexamer primers, deoxy nucleotides and MultiScribe RT (Thermo Fisher Scientific) using the following cycling program: 10 min at 25°C, followed by 120 min at 37°C, then by 5 min at 85°C.

For quantitative real-time PCR, the following QuantiTect Primer Assays (Qiagen) and KiqStart primers (Sigma) were used (all *Rattus norvegicus*): *Acta2* (QT01615901), *B2m* (QT00176295), *Bmp2* (R1_Bmp2), *Bmpr2* (R_Bmpr2_1), *Cnn1* (QT01081115), *Gusb* (R1_Gusb), *Id1* (QT00374220), *Il6* (QT00182896), *Pecam1* (QT02562581), *Serpine1* (QT00189994), *Snai1* (QT00380331), *Snai2* (QT00404866), *Twist1* (QT01290149), *Tagln* (QT00188769), *Tbp* (R1_Tbp), *Vcam1* (QT00178500). The following primers were obtained from Integrated Data Technologies (IDT, Coralville, IA): human *BMP2* (forward: 5’-CTA CCA GAA ACG AGT GGG AAA -3’; reverse: 5’-GAA GCT CTG CTG AGG TGA TAA A-3’. The following QuantiTect primers (Qiagen) were used: human *ID1* (Hs_ID1_va.1_SG) and the housekeeping gene human *B2M* (Hs_B2M_1_SG). The amplification was performed using CFX384 Touch qPCR system using iTaq Universal SYBR Green Supermix (Biorad, Hercules, CA). The cycling conditions were as follows: Preincubation for 30 sec at 95°C, then Amplification (45 cycles with 5 sec at 95°C and 30 sec at 60°C). After the last amplification, a melting curve was generated by stepwise increasing the temperature from 65°C to 95°C in

0.5°C increments every 5 s to ensure the specificity of the amplification reaction. The values were calculated according to the mathematical model published by Pfaffl M [12] by normalization against the geometric mean of *B2m, Gusb and Tbp* (rat) or against *B2M* (human) as housekeeping genes. Values were expressed as n-fold of control samples.

Protein isolation and Western blots

Whole cell protein lysate was isolated as published previously using RIPA buffer (Sigma Aldrich) [9,11]. Western blots were performed as published [9,11] and membranes were incubated overnight with the following antibodies in blocking buffer (5% dry milk/PBS/0.1% Tween 20): β-actin (A5441, Millipore Sigma, loading control), BMPR2 (BD Biosciences, San Jose, CA, 612292), cleaved caspase-3 (Cell Signaling #9661), P-Smad2 (Cell Signaling, #3101), P-Smad1/5/9 (Cell Signaling #9511).

References

1. Wu, D. & Yotnda, P. Induction and testing of hypoxia in cell culture. *J Vis Exp* (2011).
2. Zudaire, E., Gambardella, L., Kurcz, C. & Vermeren, S. A computational tool for quantitative analysis of vascular networks. *PLoS One* **6**, e27385 (2011).
3. Schindelin, J. et al. Fiji: an open-source platform for biological-image analysis. *Nat Methods* **9**, 676-682 (2012).
4. Heiss, M. et al. Endothelial cell spheroids as a versatile tool to study angiogenesis in vitro. *FASEB J* **29**, 3076-3084 (2015).
5. Alvarez, D. F. et al. Lung microvascular endothelium is enriched with progenitor cells that exhibit vasculogenic capacity. *Am J Physiol Lung Cell Mol Physiol* **294**, L419-30 (2008).

6. Farkas, D. et al. Nuclear factor kappaB inhibition reduces lung vascular lumen obliteration in severe pulmonary hypertension in rats. *Am J Respir Cell Mol Biol* **51**, 413-425 (2014).
7. Farkas, D. et al. CXCR4 inhibition ameliorates severe obliterative pulmonary hypertension and accumulation of C-kit(+) cells in rats. *PLoS One* **9**, e89810 (2014).
8. Farkas, L. et al. VEGF ameliorates pulmonary hypertension through inhibition of endothelial apoptosis in experimental lung fibrosis in rats. *J Clin Invest* **119**, 1298-1311 (2009).
9. Farkas, D. et al. Toll-like Receptor 3 Is a Therapeutic Target for Pulmonary Hypertension. *Am J Respir Crit Care Med* **199**, 199-210 (2019).
10. Oka, M. et al. Rho kinase-mediated vasoconstriction is important in severe occlusive pulmonary arterial hypertension in rats. *Circ Res* **100**, 923-929 (2007).
11. Bhagwani, A. R. et al. Endothelial cells are a source of Nestin expression in Pulmonary Arterial Hypertension. *PLoS One* **14**, e0213890 (2019).
12. Pfaffl, M. W. A new mathematical model for relative quantification in real-time RT-PCR. *Nucleic Acids Res* **29**, e45 (2001).

SUPPLEMENTAL FIGURE LEGENDS

Supplemental Figure S1: Representative staining for Podocalyxin (PODXL), an endothelial cell marker, and CD117. Representative pseudo coloured optical sections (confocal microscopy) for PODXL (green pseudo colour) and CD117 (red pseudo colour) staining in pulmonary arteries from control, and in intima lesions and plexiform lesions from PAH patients. Arrows demonstrate the $PODXL^+$ $CD117^+$ cells ($CD117^+$ endothelial cells) which are rare in control pulmonary arteries, but frequent in pulmonary arterial lesions from PAH patients. Nuclear staining with DAPI (blue pseudo colour). Scale bar: 25 µm.

Supplemental Figure S2. Characterization of control rat ECs isolated from the $CD117^-$ lung cell pool. (**A**) DIC image of cultured cells, (**B**) DIC image of 24h 2D matrigel tube formation assay demonstrates formation of tubes, albeit low number of branches. (**C**) Binding of *Griffonia simplicifolia* (*G.s.*) lectin (red pseudo colour) indicates microvascular endothelial phenotype - image obtained by confocal microscopy. (**D**) Tube formation in 3d fibrin assays - image shows maximum intensity projection of Z-stack obtained by confocal microscopy. Actin filaments were stained with rhodamine-labeled phalloidin (red pseudo colour). Nuclear staining in (C-D) with DAPI (blue pseudo colour) (**E**) Representative flow cytometry analysis of surface markers demonstrating expression of endothelial surface markers CD144, VEGFR2 and CD105. Hematopoietic surface markers CD45 and CD11b/c were not detected. Grey: isotype, Dark grey/black: specific antibody staining. (C-D): nuclear counterstaining with DAPI. Scale bars: 50 µm (C), 100 µm (A), 200 µm (D), 500 µm (B).

Supplemental Figure S3: Inhibition of CD117 ligand stem cell factor (SCF) reduces cell viability and angiogenic tube formation. (**A**) Representative stitched differential

interference contrast (DIC) images of CD117$^+$ EC clones treated with control antibody (ab, goat serum) or anti-SCF ab (10 µg/ml) for 24 h during tube formation assay on matrigel. Scale bar: 25 µm. (**B**) CellTiter Glow indicates reduced cell viability following 24h of treatment with anti-SCF antibody. (**C**) Quantification of total network length in matrigel assay and treatment with control ab or anti-SCF ab. *$P<0.05$, **$P<0.01$. n=3, mean+SEM.

Supplemental Figure S4: Transformation of EC clones to smooth muscle-like cells. (**A**) Representative DIC images showing EC clones cultured for 7 days in complete EGM-2MV medium, MEM medium (with 10% FBS and antibiotics) and MEM medium supplemented with TGF-β1 (5 ng/ml) and PDGF-B (25 ng/ml). The experiments indicate that changing to a more basal medium resulted in switch to a mesenchymal phenotype. Scale bar: 100 µm. (**B**) mRNA expression of endothelial markers *Pecam1* (CD31) and *Vcam1*, of smooth muscle markers *Acta2*, *Cnn1* and *Tagln*, and mesenchymal transition transcription factors *Snai1* (Snail), *Snai2* (Slug) and *Twist1*. n=3-5 per group. (**C**) Representative DIC images showing EC clones cultured for 7 days in MEM medium and MEM medium supplemented with TGF-β1 (5 ng/ml). Scale bar: 100 µm. (**D**) mRNA expression of endothelial markers/TGF-β downstream target *Vcam1*, of smooth muscle markers *Acta2*, *Cnn1* and *Tagln*, and mesenchymal transition transcription factors *Snai1* (Snail), *Snai2* (Slug) and *Twist1*. n=6-9 per group. mean+SEM. *$P<0.05$, **$P<0.01$, ***$P<0.001$.

Supplemental Figure S5. Mesenchymal lineage differentiation experiments. (**A**) 28 days of culture in adipogenic differentiation medium caused massive reduction in cell density for EC clones without production of lipid droplets as shown by Oil Red O staining. In contrast, bone marrow mesenchymal stem cells (BM-MSCs) produced lipid droplets (bright red

staining) and served as positive control for the lineage differentiation assays. BM-MSCs (-) is negative control without adipogenic medium. (**B**) 28 days of culture in osteogenic medium induced formation of conglomerates with Ca^{2+} complex deposition (bright red staining), similar to BM-MSCs (+). BM-MSCs (-) is negative control without osteogenic medium. Scale bars: 25 µm.

Supplemental Figure S6: Injection of EC clones fails to induce occlusive pulmonary arterial remodelling under conditions of normoxia. (**A**) Representative images of vWF IHC demonstrate vWF staining (dark brown) in pulmonary arteries from rats exposed to normoxia+vehicle and normoxia+$CD117^{+}$ EC clones. Scale bar: 100 µm. (**B**) RVSP shows no difference between rats exposed to normoxia+vehicle and normoxia+$CD117^{+}$ EC clones. n=3 per group.

Supplemental Figure S7: Injection of $CD117^{+}$ ECs (without clonal expansion) fails to induce occlusive pulmonary arterial remodeling under conditions of normoxia and hypoxia, even when injections were performed once a week for 3 weeks (total 3 injections). (**A**) Representative immunofluorescence stainings for vWF (green pseudo colour) and α-SMA (red pseudo colour) demonstrate normal remodeling under chronic hypoxia, but no hemodynamically relevant occlusion. Nuclear staining with DAPI (blue pseudo colour) (**B**) RVSP shows comparable levels to normoxia alone and chronic hypoxia alone. n=2 per group.

Supplemental Figure S8: Transplanted $EGFP^{+}$ cells were not identified by GFP antibody staining (green pseudo colour) in the right ventricle of rats exposed to chronic hypoxia for 21 days. (**A**) Stitched overview image shows no GFP^{+} cells detected in RV of cHx+EC

clone rats. The areas with enhance green background fluorescence are small folding areas of the tissue from sectioning. (**B**) No GFP$^+$ cells were detected on higher detail images and in co-staining with DAPI (**C**), small positive dots (arrows) were identified as artefacts, but not as positive cells. (**D**) Positive and negative controls for GFP staining: positive control was lung tissue derived from EGFP transgenic rats and for negative control, the primary antibody was omitted. Scale bars: 200 µm (A), 100 µm (B, D).

Supplemental Figure S9: Representative flow cytometry for GFP$^+$ cells in one lobe of the right lung from chronic hypoxia+EC clones at day 21. The dot plot on the left shows staining with secondary antibody (ab) only, whereas the dot plot on the right indicates staining with anti-GFP primary ab and secondary ab. The GFP$^+$ gate is shown in both dot plots.

Supplemental Figure S10: Real-time PCR from the lungs of rats treated with cHx+CD117$^-$ ECs and cHx alone. There was a trend towards elevated *Bmp2* expression, whereas *Bmpr2* and *Il6* expression were reduced. Mean+SEM. n=3 per group. **P<0.01, ***P<0.001.

Supplemental Figure S11: Effect of exposure of CD117$^+$ EC clones to hypoxia (5% O_2) for 48h on BMP pathway activity and Edn1 expression. (**A**) mRNA expression of *Bmp2, Bmpr2* and *Id1* was reduced following exposure to hypoxia for 48h. mean+SEM. n=4-5/group. *P<0.05, **P<0.01. (**B**) Representative Western blots demonstrate reduced Smad1/5/9 phosphorylation in EC clones after 48h of exposure to hypoxia in comparison to normoxia. β-actin was used as loading control.

Supplemental Figure S12: Full-length Western blots for Figure 3G (A), Figure 5F (B) and Supplemental Figure S10 (C). Note that two different exposure times are presented for each Western blot. The cropped areas are shown by red rectangles.

SUPPLEMENTARY FIGURES

Figure S1

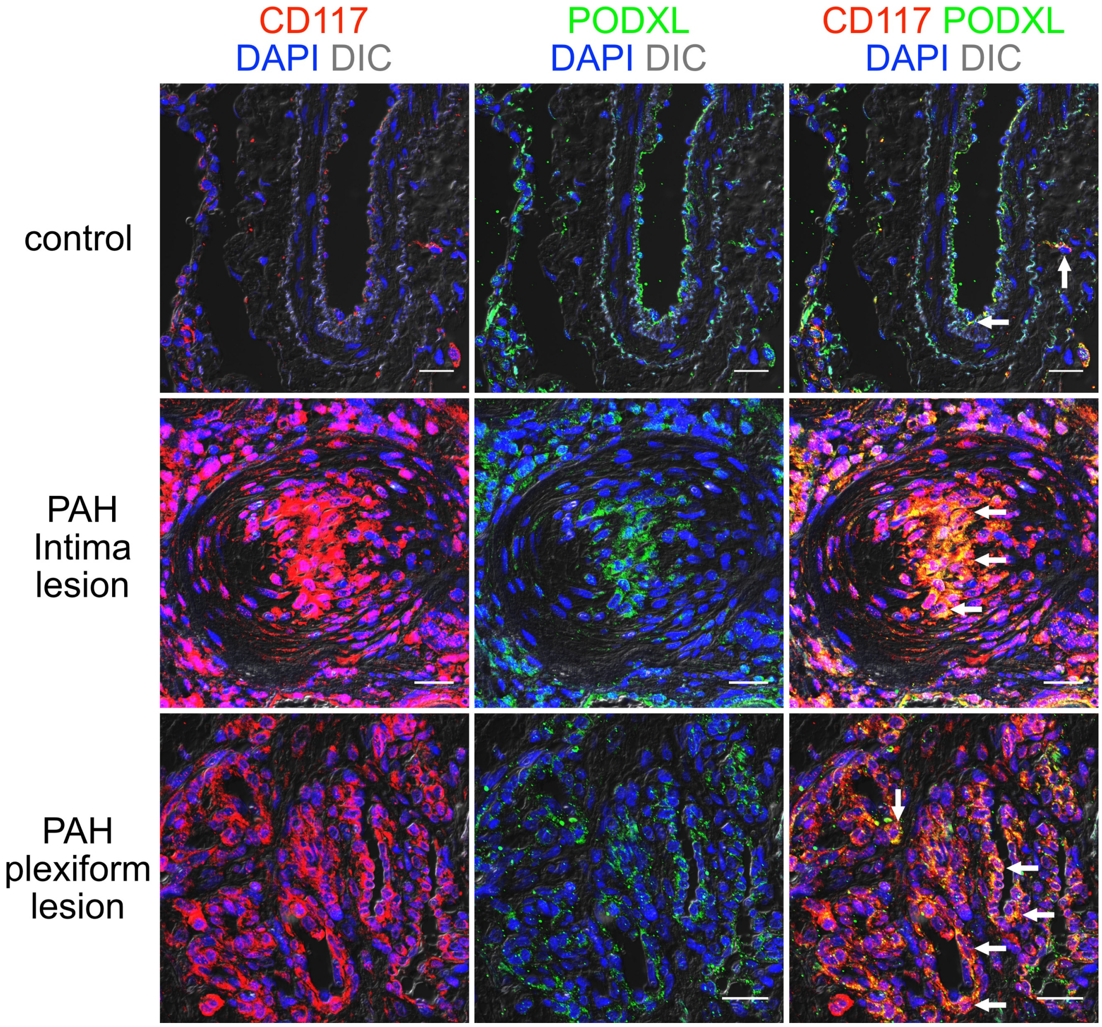

Figure S2

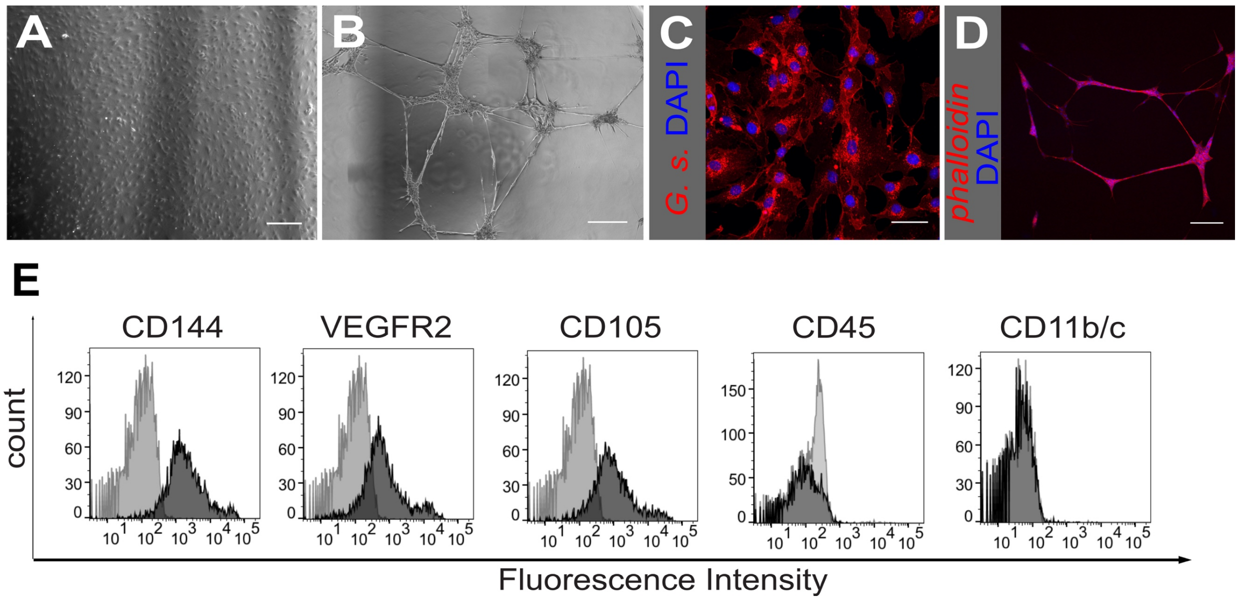

A
B
C
G. s. DAPI
D
phalloidin DAPI
E
CD144
VEGFR2
CD105
CD45
CD11b/c
count
Fluorescence Intensity

Supplemental Figure S3

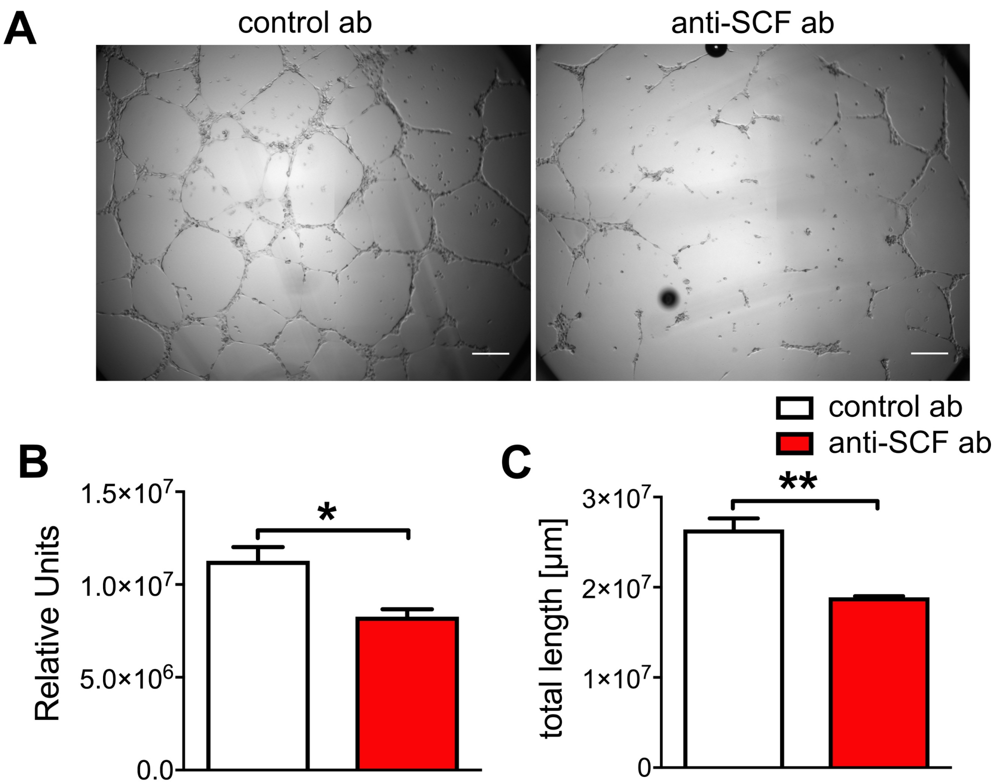

Supplemental Figure S4

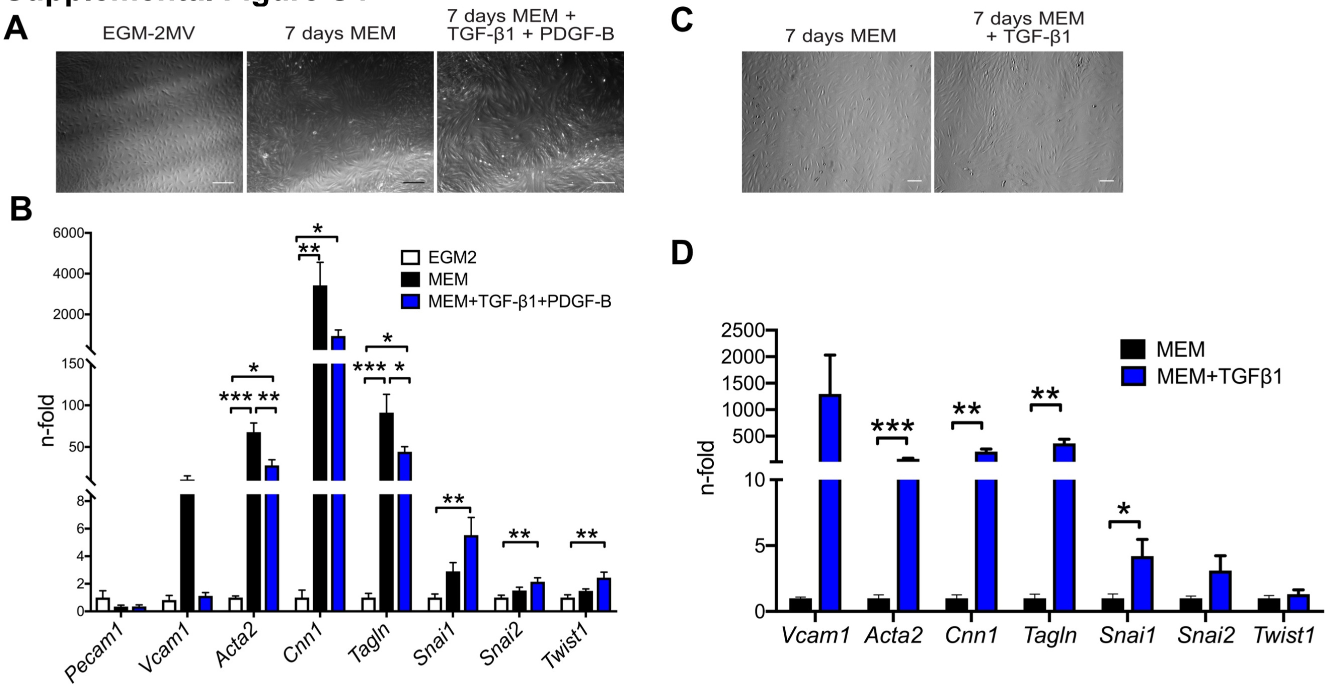

Supplemental Figure S5

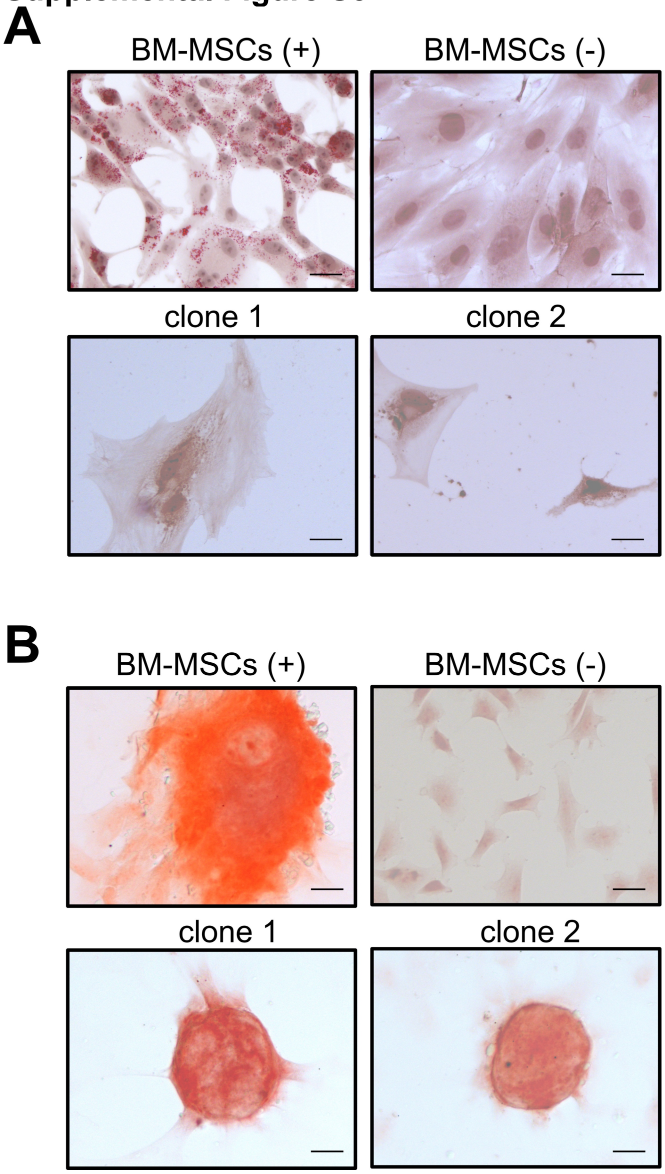

Supplemental Figure S6

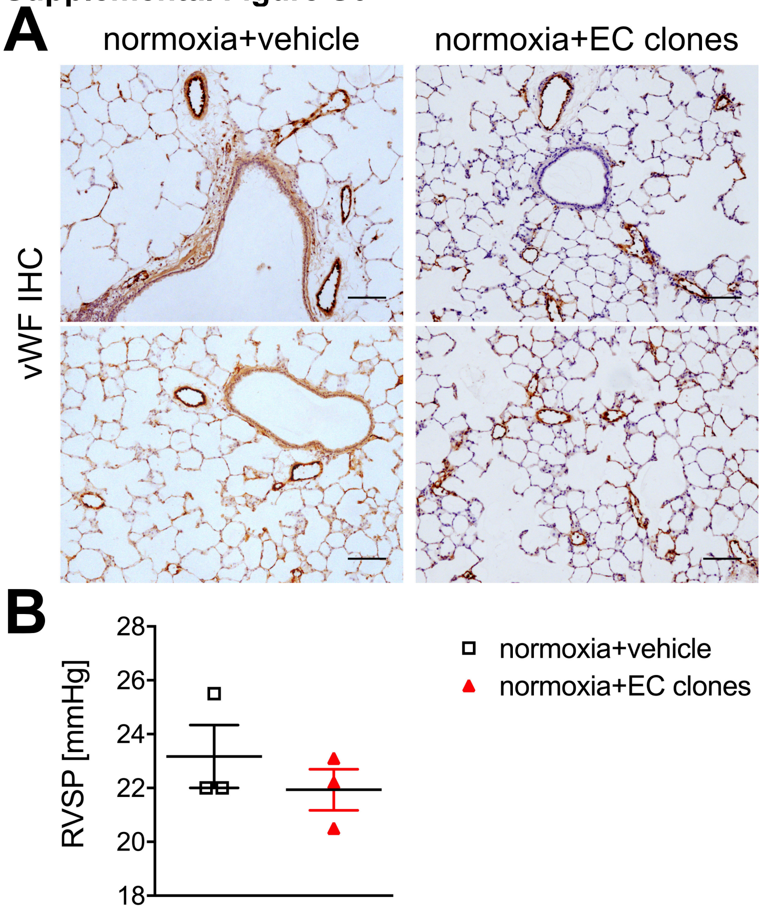

Supplemental Figure S7

A

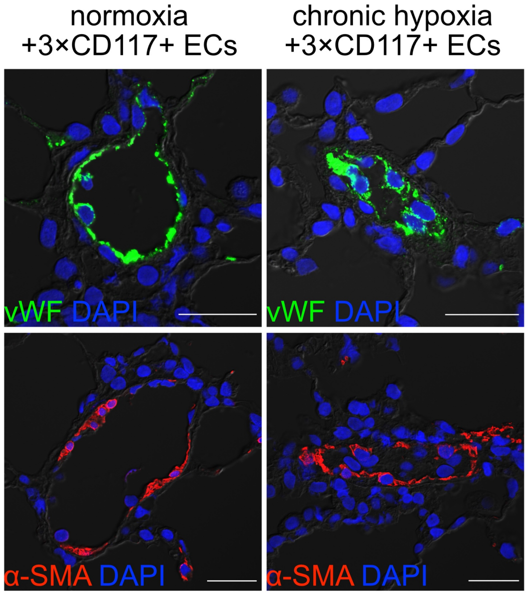

B

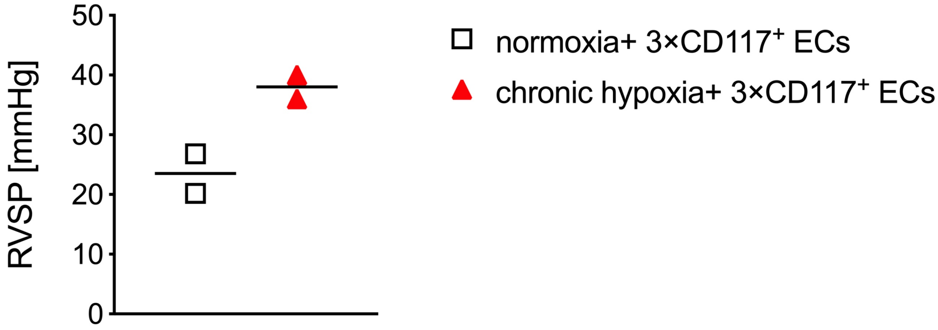

Supplemental Figure S8

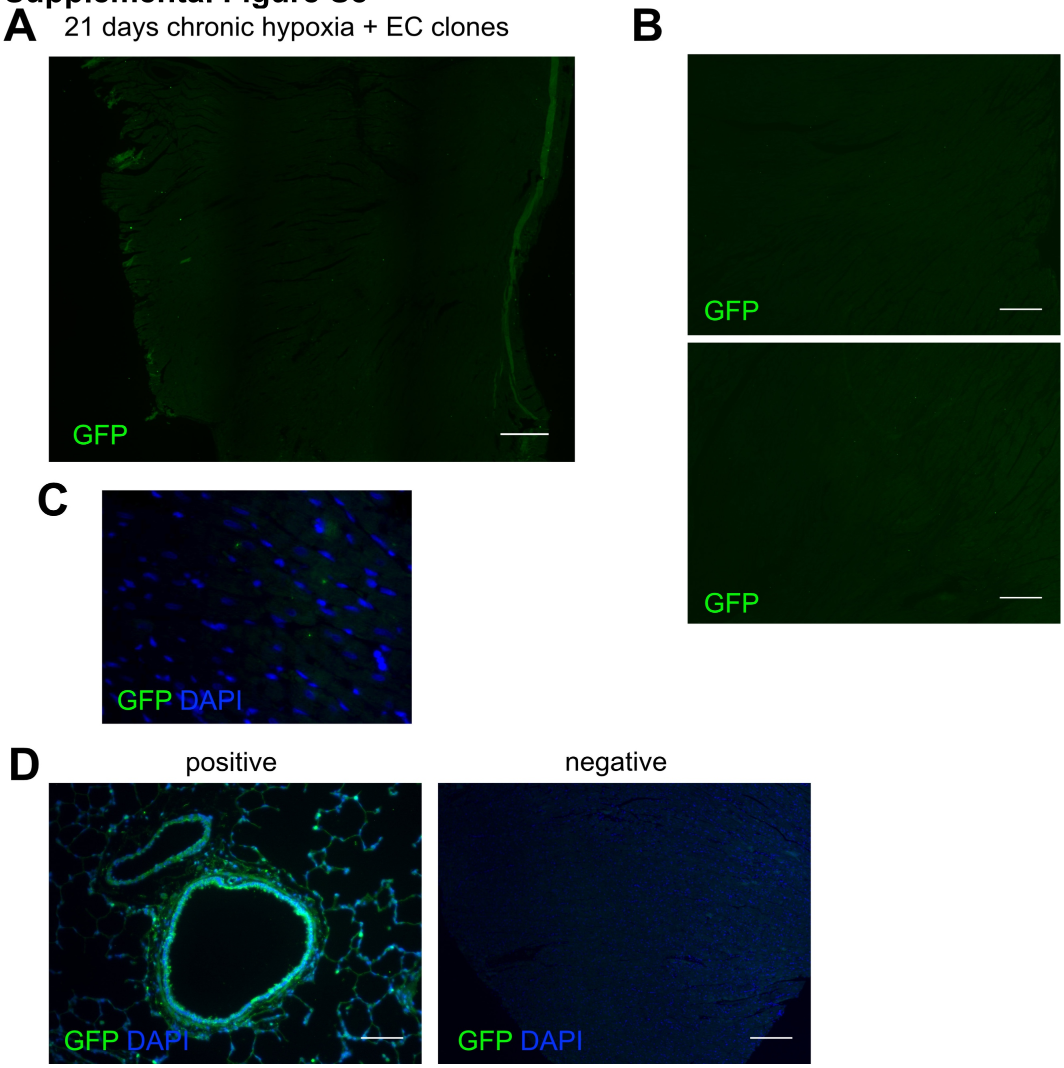

Supplemental Figure S9

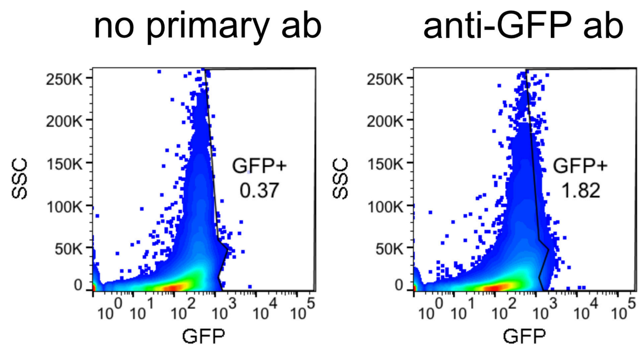

Supplemental Figure S10

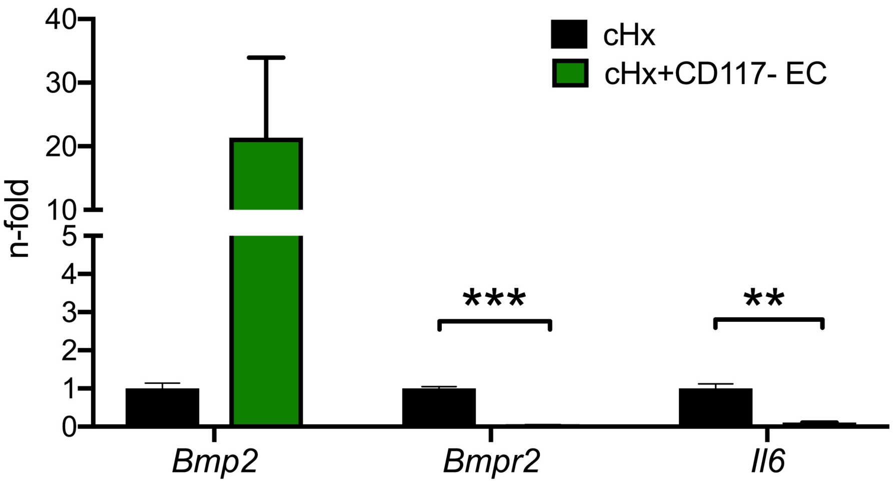

Supplemental Figure S11

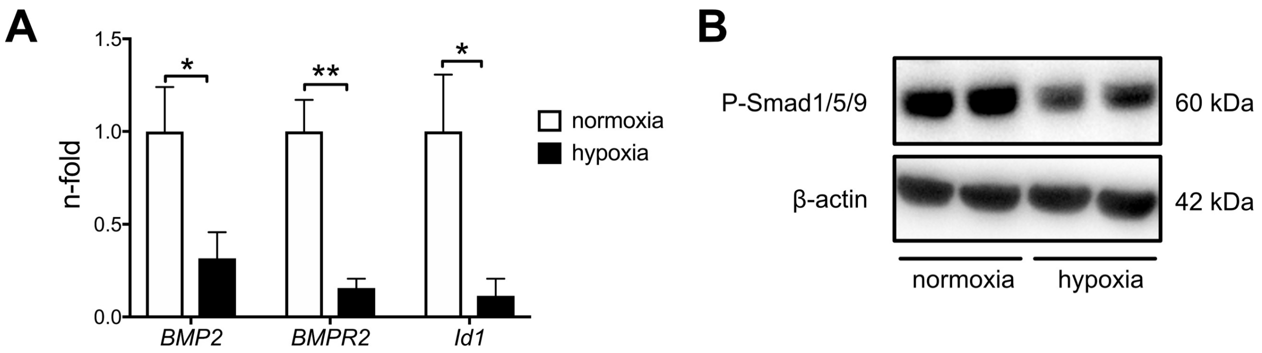

Supplemental Figure S12

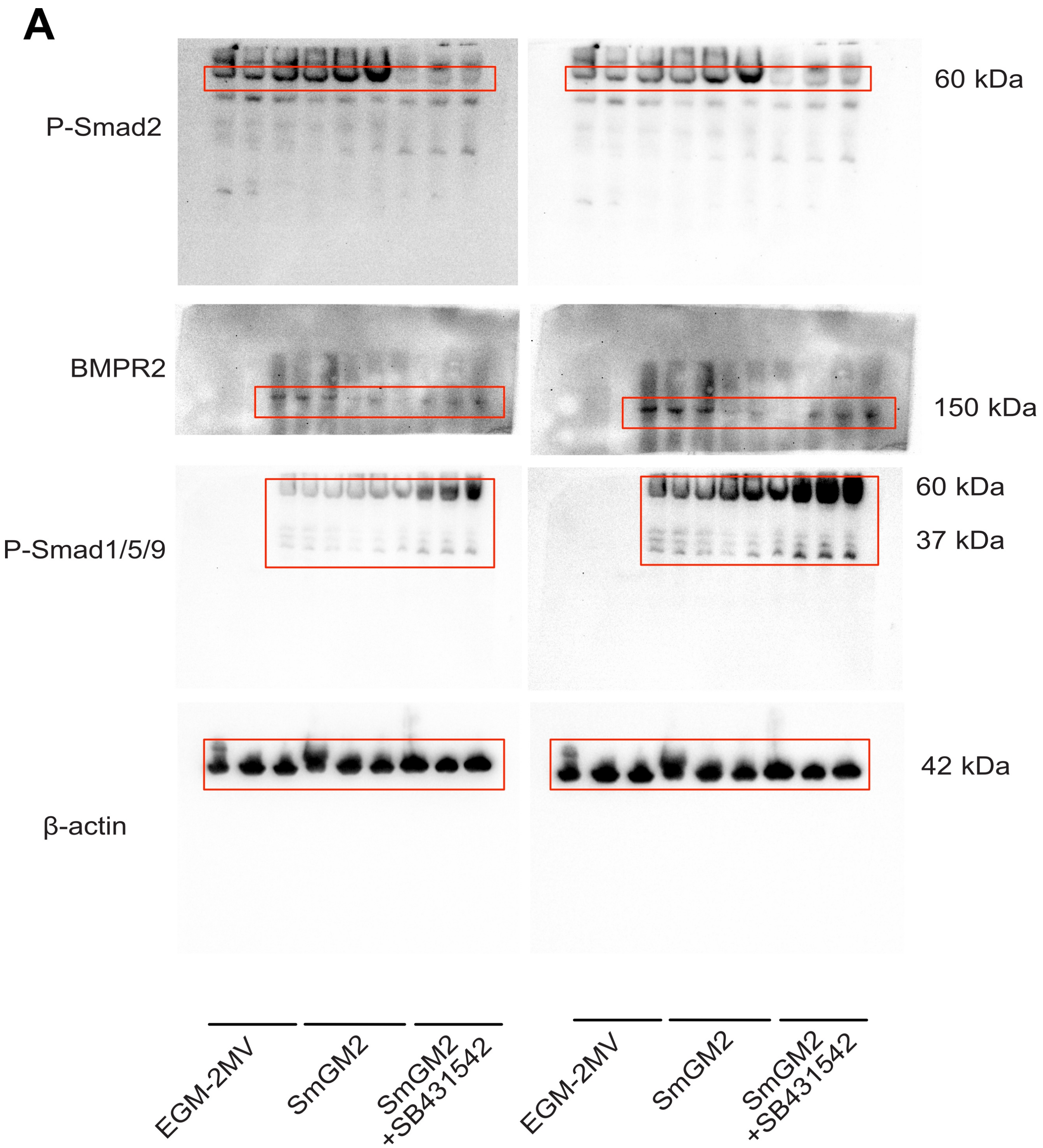

B

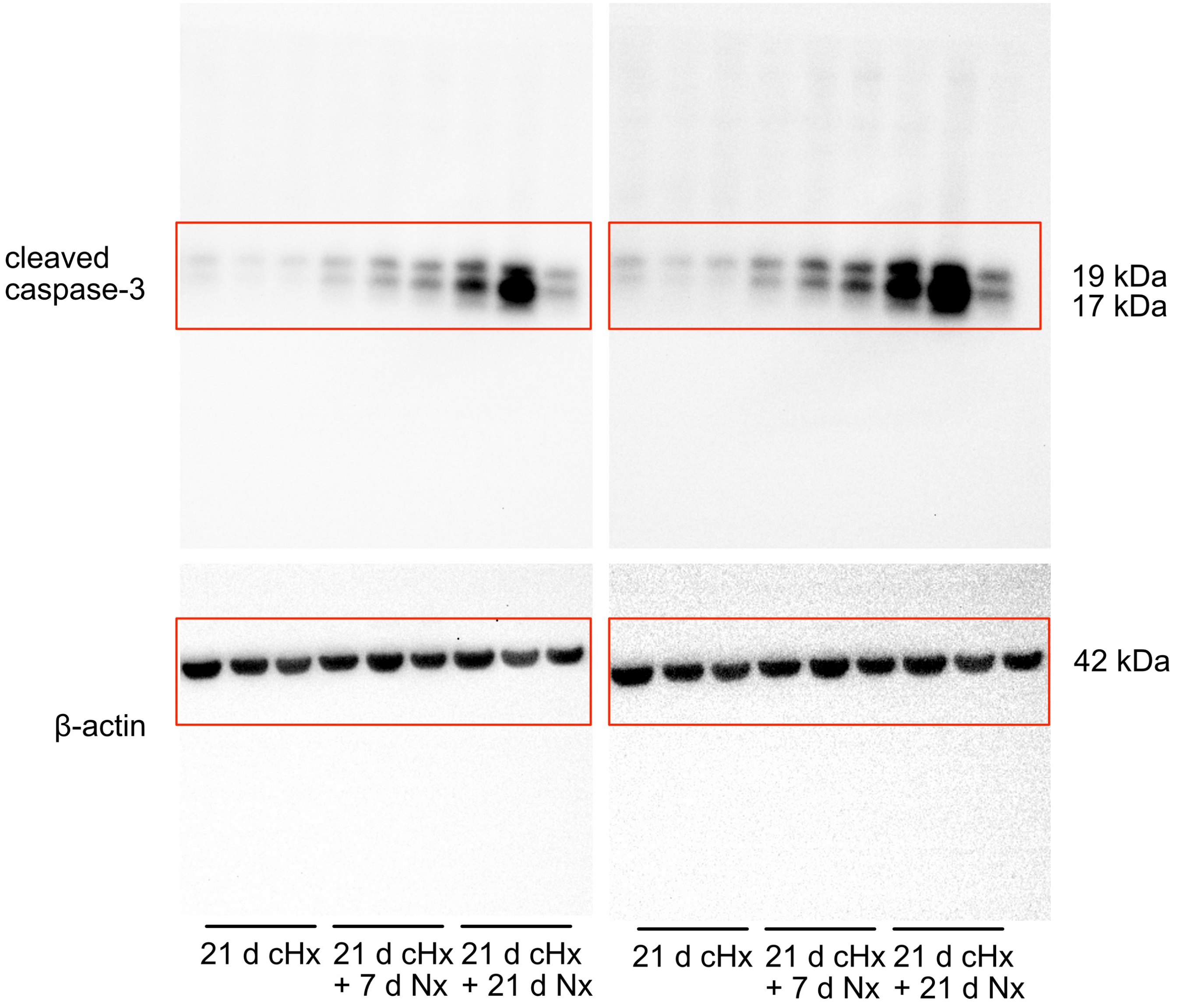
cleaved
caspase-3
19 kDa
17 kDa
β-actin
42 kDa
21 d cHx
21 d cHx
+ 7 d Nx
21 d cHx
+ 21 d Nx
21 d cHx
21 d cHx
+ 7 d Nx
21 d cHx
+ 21 d Nx

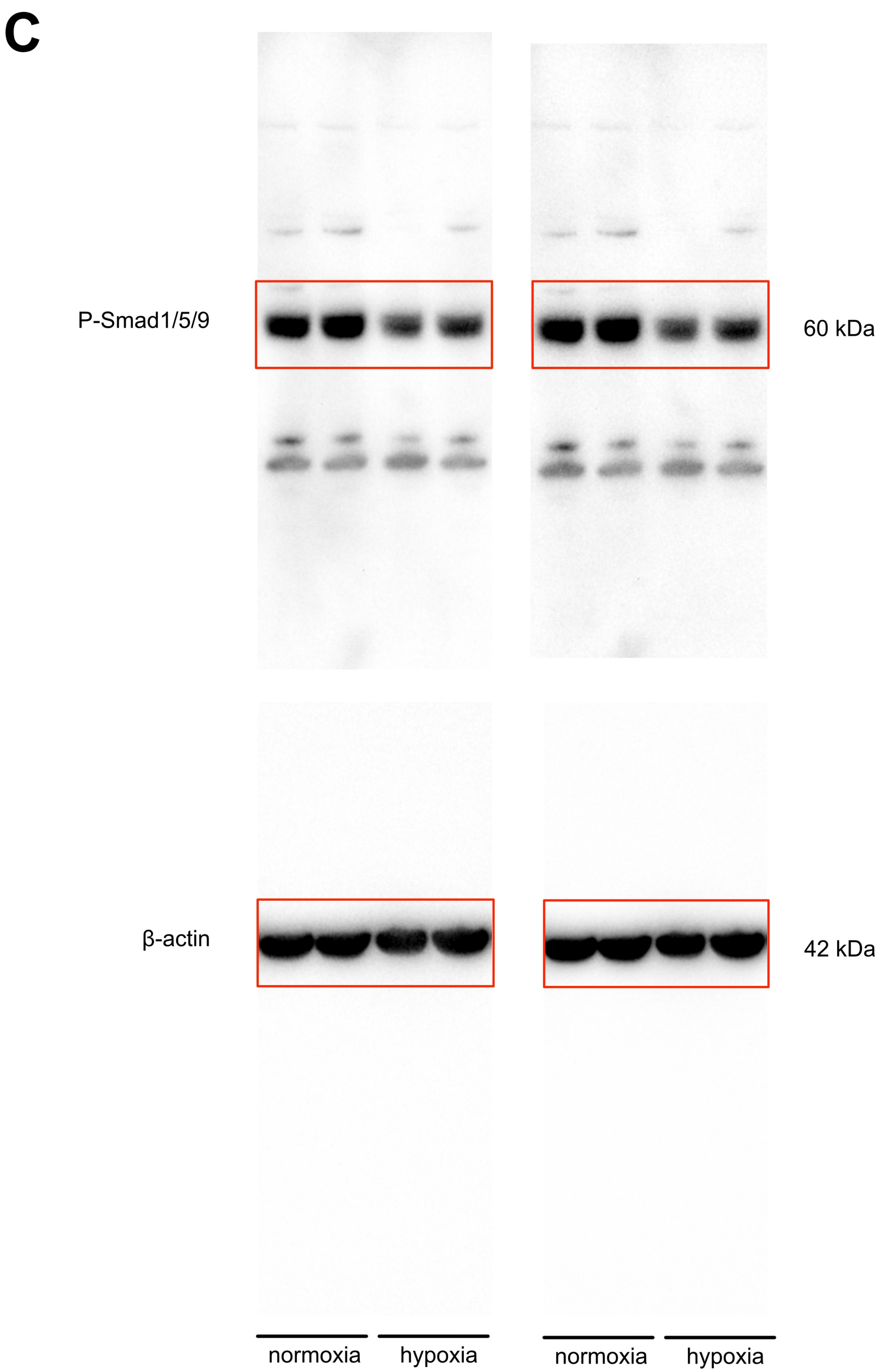
C
P-Smad1/5/9
60 kDa
β-actin
42 kDa
normoxia
hypoxia
normoxia
hypoxia

Supplementary Table S1: Summary of the pathway/disease clusters with significant changes between EC clones and control ECs. Changes in the major canonical pathways for EC clones (n=4) vs. control ECs (from CD117^{-} lung cells, n=3) are shown. The pathways indicated are scored using Fisher's Exact Test p-value. Predicted changes in gene activity are expressed as z score with positive z score indicating increased activation and negative z score indicating decreased activation. Where no z score is provided, the results did not allow for prediction of activation. The stringent cut-off values of $p \leq 0.05$ and a fold-change (FC) cut-off of ±10 were applied to this analysis.
